# The Biological Disease-Modifying Antirheumatic Drugs and the Risk of Cardiovascular Events: A Systematic Review and Meta-Analysis

**DOI:** 10.1155/2021/7712587

**Published:** 2021-08-31

**Authors:** Suiyuan Hu, Chu Lin, Xiaoling Cai, Xingyun Zhu, Fang Lv, Lin Nie, Linong Ji

**Affiliations:** ^1^Department of Endocrinology and Metabolism, Peking University People's Hospital, Beijing, China; ^2^Department of Endocrinology and Metabolism, Beijing Airport Hospital, Beijing, China

## Abstract

**Objective:**

To assess the association between the use of biological disease-modifying antirheumatic drugs (bDMARDs) and the risk of cardiovascular events in patients with systemic inflammatory conditions.

**Methods:**

Eligible cohort studies or randomized controlled trials (RCTs) from inception to January 2021 were included. Pooled odds ratios (ORs) with 95% confidence intervals (CIs) for cardiovascular outcomes were calculated in the fixed- and random-effects model accordingly. Associated factors with risks of cardiovascular events were also studied in sensitivity analyses and metaregression analyses.

**Results:**

Compared with non-bDMARD users, the risks of myocardial infarction (MI) (OR = 0.74, 95% CI, 0.63 to 0.87), heart failure (OR = 0.84, 95% CI, 0.74 to 0.95), cardiovascular (CV) death (OR = 0.62, 95% CI, 0.40 to 0.95), all-cause mortality (OR = 0.64, 95% CI, 0.58 to 0.70), and 3P-MACE (composite endpoint of MI, stroke, and CV death) (OR = 0.69, 95% CI, 0.53 to 0.89) were significantly reduced in bDMARD users, which were mainly driven by the risk reduction in patients with rheumatoid arthritis (RA). TNF-*α* inhibitors exhibited consistent benefits in reducing the risks of MI, heart failure, CV death, all-cause mortality, and 3P-MACE. Moreover, the risks of heart failure, CV death, all-cause mortality, and 3P-MACE were significantly reduced in bDMARD users with follow-up over one year.

**Conclusions:**

The use of bDMARDs might be associated with the reduced risks of CV events, especially in patients with RA. The CV events might be less frequent in bDMARD users with TNF-*α* inhibitors or follow-up over one year. More investigations are needed to validate conclusions.

## 1. Introduction

Cardiovascular disease (CVD) is the leading cause of death worldwide [1]. With accumulated evidence [2–5], the presence of chronic inflammation was proposed to play an important role in cardiovascular (CV) risk. It was suggested that inflammation might modify traditional CV risk factors such as lipids [2], which were involved in the pathogenesis of atherosclerosis [3], promoting endothelial dysfunction and microvascular disease [4, 5]. Therefore, this spurred researchers to seek therapeutics that target inflammation to reduce CV events. Inspiringly, encouraging results from the recent anti-inflammatory trials in population with CVD, such as CANTOS [6] and LoDoCo2 [7], indicated that anti-inflammatory treatments might provide CV protective effects.

Meanwhile, several systemic inflammatory diseases have been found to be associated with excess CV risk. It was revealed that cardiovascular disease accounted for the largest proportion of excess mortality in rheumatoid arthritis (RA) [8]. What is more, individuals with systemic lupus erythematosus (SLE) were observed to have increased incidence of ischemic stroke or myocardial infarction (MI) [9], which were also found in patients with psoriasis and other inflammatory diseases [10]. It is putative that the excess CV risk in many inflammatory diseases might be mediated through the persistent inflammation and oxidative stress [11].

As a fundamental therapy for systemic inflammatory diseases, anti-inflammatory agents were commonly used, among which biological disease-modifying antirheumatic drugs (bDMARDs) developed rapidly and were gradually applied in clinical management in the past two decades, for their potent anti-inflammatory effects and specific targets. Besides the prompt and sustained efficacy in disease remission and the safety of bDMARDs in systemic inflammatory conditions [12–14], cardioprotective effects were found in tumor necrosis factor-*α* (TNF-*α*) inhibitor treatment [15–18] in patients with RA and psoriasis and were also suggested in certain biological agents, such as tocilizumab and rituximab [19–21]. Meanwhile, some agents like adalimumab and ustekinumab might be associated with the increased risk of cardiovascular events [18, 22–24] Therefore, the cardiovascular effects of bDMARDs have yet to be definitive.

In order to reveal the association between the use of bDMARDs and the risks of cardiovascular events in patients with systemic inflammatory conditions, we designed and performed a systematic review and meta-analysis, with the aim to evaluate whether treatments with bDMARDs would reduce the risk of cardiovascular events in patients with systemic inflammatory conditions.

## 2. Methods

### 2.1. Study Design and Electronic Literature Search Strategy

According to the recommendations from the Cochrane Handbook for Systematic Reviews for meta-analysis, we conducted systematic searches in PubMed, Medline, Embase, the Cochrane Central Register of Controlled Trials, and Clinicaltrial.gov for studies of bDMARD published from inception to January 2021. The search strategies included the following search terms: bDMARD, biological therapy, biological agent, RA, SLE, psoriasis, cardiovascular risk, cardiovascular event, cardiovascular disease, TNF-*α* inhibitor, infliximab, etanercept, adalimumab, certolizumab, golimumab, IL (receptor) inhibitor, anakinra, tocilizumab, CD17 inhibitor, secukinumab, brodalumab, ixekizumab, CD23 inhibitor, risankizumab, tildrakizumab, guselkumab, CD22 inhibitor, epratuzumab, CD12/23inhibitor, briakinumab, ustekinumab, CD20 antibody, CD20 inhibition, rituximab, belimumab, blisibimod, atacicept, tabalumab, sifalimumab, CD80/86 inhibition, abatacept, interferon receptor antibody, anifrolumab, observational study, cohort study, and RCT. We also screened reference lists of relevant articles in order not to miss any possibly eligible study.

### 2.2. Selection of Articles, Data Extraction, and Quality Assessment

The inclusion criteria for the meta-analysis were as follows: (1) cohort studies or randomized controlled trials (RCTs) with presented outcomes of cardiovascular events between bDMARD users and non-bDMARD users; (2) studies conducted in systemic inflammatory conditions including RA, SLE, and psoriasis. The exclusion criteria were as follows: (1) studies without presented outcomes of cardiovascular events; (2) studies with bDMARDs in both treatment arms; (3) studies with participants less than 18 years old. Two investigators (SH and CL) performed the study selection independently. A third investigator (FL) reexamined the selected results. Every disagreement would be pointed out and resolved by a joint discussion until a consensus was reached.

Study data was abstracted from eligible RCTs and cohorts by two investigators (SH and CL), including first author, publication year, study design, numbers of participants, age, disease duration, drug exposure, history of CVD, incidence of cardiovascular events, and any available efficacy endpoint reported in the studies. If data on cardiovascular events could not be accessed in both original articles and supplementary materials, the investigators would search from Clinicaltrial.gov website with the unique registered NCT number. Qualities of the observational studies were evaluated by using the Newcastle-Ottawa Scale, and qualities of RCTs were evaluated by using the Cochrane risk of bias tool. A third investigator (FL) confirmed the accuracy of the abstractions and study quality evaluation. Any disagreement would be resolved by a joint discussion until a consensus was reached.

### 2.3. Definition of Clinical Outcomes

In this meta-analysis, we set up two composite CV endpoints, which consisted of a three-point major adverse cardiovascular event (3P-MACE, including myocardial infarction (MI), stroke, and CV death) and a four-point major adverse cardiovascular event (4P-MACE, including 3P-MACE and heart failure). In addition, we separately analyzed MI, stroke, CV death, heart failure, and all-cause mortality as individual CV outcomes.

### 2.4. Statistical Analysis

Results of the meta-analysis were presented by the odds ratio (OR) along with the 95% confidence interval (CI). Higgins *I*^2^ statistics were used to evaluate the heterogeneity between different studies, when an *I*^2^value > 50% indicates a high level of heterogeneity. A fixed-effects model was used for low level of heterogeneity, and a random-effects model was used for high level of heterogeneity. Metaregression analyses were performed to evaluate whether age, sex, disease duration, concomitant medication, and efficacy endpoints were associated with the risk of cardiovascular events. Statistical analyses were performed by the Review Manager statistical software package (Version 5.3, Nordic Cochrane Center, Copenhagen, Denmark). Metaregression analyses were performed by STATA, version 11.0 (STATA, College Station, TX, USA). All statistical analyses with *P* value < 0.05 were considered statistically significant.

## 3. Results

### 3.1. Characteristics of Included Studies

**Figure 1:**
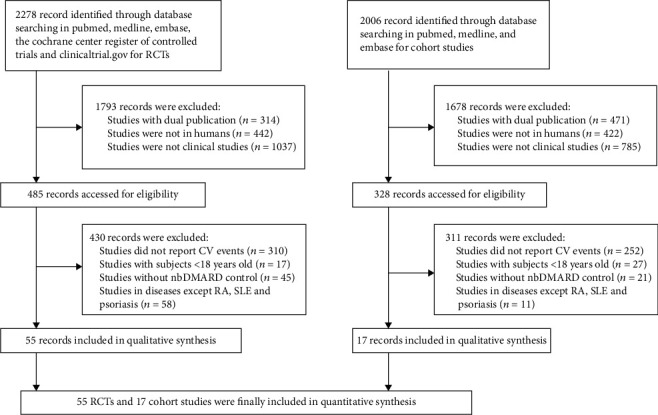
Flowchart of included studies.

In all, 72 studies were included (38 studies with RA, 21 studies with psoriasis, and 13 studies with SLE), with 55 RCTs and 17 cohort studies, respectively (Figure 1). The enrolled systemic inflammatory conditions were RA, psoriasis, and SLE. Baseline characteristics of included studies are summarized in Table S1-S2. This meta-analysis was registered in the PROSPERO platform as CRD42020207140.

The risk of bias for RCT was systematically evaluated by the Cochrane tool (Table S3). There were 14 RCTs with unclear risk of selection bias (random sequence generation), 1 RCT with high risk and 7 RCTs with unclear risk of selection bias (allocation concealment), 2 RCTs with high risk and 1 RCT with unclear risk of performance bias, and 19 RCTs with high risk and 2 RCTs with unclear risk of attrition bias. The included cohort studies were of relatively high quality as Newcastle-Ottawa Scale suggested (Table S4). The funnel plots for individual and composite CV endpoints generally displayed even distributions (Figure S1).

### 3.2. The Association between the Use of bDMARDs and the Risk of CV Events

**Figure 2:**
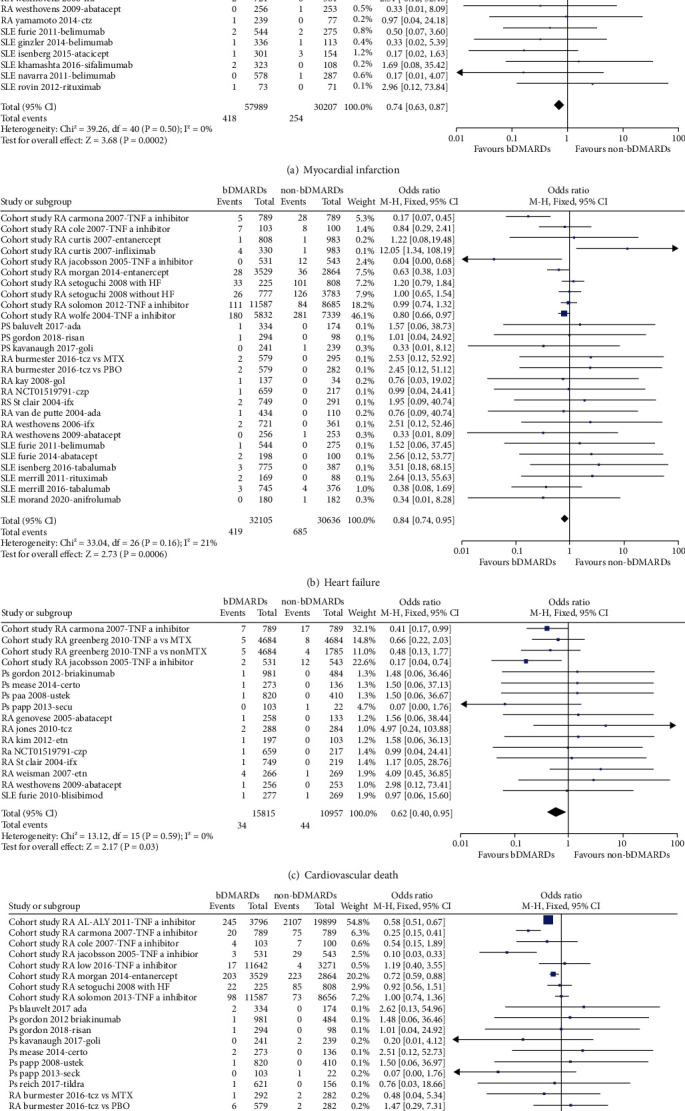
The associations between the use of bDMARD and cardiovascular endpoint.

Overall, compared with non-bDMARDs users, the risks of MI (OR = 0.74, 95% CI, 0.63 to 0.87, *I*^2^ = 0%), heart failure (OR = 0.84, 95% CI, 0.74 to 0.95, *I*^2^ = 21%), CV death (OR = 0.62, 95% CI, 0.40 to 0.95, *I*^2^ = 0%), and all-cause mortality (OR = 0.64, 95% CI, 0.58 to 0.70, *I*^2^ = 38%) were significantly reduced in bDMARD users (Figure 2), which were mainly driven by the results of cohort studies (Tables 1–4).

When stratified by the systemic inflammatory conditions, subgroup analyses showed significant reduction in risks of MI (OR = 0.74, 95% CI, 0.63 to 0.87, *I*^2^ = 31%), heart failure (OR = 0.83, 95% CI, 0.73 to 0.95, *I*^2^ = 41%), CV death (OR = 0.60, 95% CI, 0.38 to 0.96, *I*^2^ = 4%), and all-cause mortality (OR = 0.64, 95% CI, 0.49 to 0.85, *I*^2^ = 63%) in bDMARD users with RA compared with non-bDMARD users (Tables 1–4).

When stratified by different drug categories, it was indicated that when compared with non-bDMARD users, the risks of MI (OR = 0.74, 95% CI, 0.63 to 0.88, *I*^2^ = 27%), heart failure (OR = 0.83, 95% CI, 0.73 to 0.95, *I*^2^ = 43%), CV death (OR = 0.53, 95% CI, 0.33 to 0.86, *I*^2^ = 0%), and all-cause mortality (OR = 0.63, 95% CI, 0.47 to 0.84, *I*^2^ = 66%) were significantly decreased in TNF-*α* inhibitor users. However, no statistically significant differences of the risks of CV events were found in other subtypes of bDMARDs (Tables 1–4).

When stratified by the follow-up period, compared with non-bDMARD users, the risks of MI (OR = 0.73, 95% CI, 0.61 to 0.87, *I*^2^ = 37), heart failure (OR = 0.80, 95% CI, 0.69 to 0.93, *I*^2^ = 38%), CV death (OR = 0.46, 95% CI, 0.28 to 0.77, *I*^2^ = 0%), and all-cause mortality events (OR = 0.62, 95% CI, 0.48 to 0.80, *I*^2^ = 42%) were less frequent in bDMARD users with a follow-up period longer than one year. However, such risk reductions were not observed in patients with a follow-up period less than one year (Tables 1–4).

As for history of CVD and its comorbidities, the results showed that risk of MI was mainly reduced in strata with lower percentage of previous CVD, diabetes, and dyslipidemia (Table 1). The risk of all-cause mortality was mainly reduced in strata with lower percentage of previous CVD and dyslipidemia (Table 4).

However, bDMARDs did not reduce the risk of stroke in patients with RA, SLE, and psoriasis when compared with non-bDMARD treatment. Subsequent sensitivity analyses did not reveal any significant association concerning the risk of stroke either (Table S5).

Furthermore, compared with non-bDMARDs, the use of bDMARDs was significantly associated with reduced risk of the composite endpoint of 3P-MACE in patients with systemic inflammatory conditions (OR = 0.69, 95% CI, 0.53 to 0.89, *I*^2^ = 0%). Subgroup analyses indicated the reduced risk of the composite endpoint of 3P-MACE in patients with RA (OR = 0.64, 95% CI, 0.47 to 0.88, *I*^2^ = 0%), in patients with TNF-*α* treatment (OR = 0.61, 95% CI, 0.45 to 0.84, *I*^2^ = 0%), and in patients with follow-up duration more than one year (OR = 0.59, 95% CI, 0.43 to 0.80, *I*^2^ = 0%) (Table S6). Similarly, the risk of 3P-MACE was also reduced in strata with lower percentage of previous CVD, diabetes, hypertension, and dyslipidemia. However, the risk of 4P-MACE was not significantly decreased in bDMARD users when compared with non-bDMARD users (OR = 0.99, 95% CI, 0.72 to 1.35, *I*^2^ = 0%) (Table S6).

### 3.3. Associated Factors with the Risks of CV Events in bDMARD Treatment

Data from the metaregression analysis showed that patient age was positively associated with risks of 3P-MACE (*β* = 0.278, 95% CI, 0.071 to 0.486) and 4P-MACE (*β* = 0.255, 95% CI, 0.039 to 0.471) in patients with RA who used bDMARDs (Table S7), but not in other diseases or cohort studies. No significant associations were found between the baseline characteristics of patients (sex, disease duration, body mass index, weight, ever smoking, etc.), or comorbidities (previous CV event, diabetes mellitus, hypertension, dyslipidemia, etc.), or changes in inflammatory indicators (CRP and ESR), and the risks of CV events in bDMARD users in metaregression analyses (Table S7). Additionally, no significant associations were found between the efficacy indicators such as DAS28 remission, ACR, BILAG, SRI response rate, SLEDAI, or PASI and the risks of CV events in bDMARD users (Table S7).

## 4. Discussion

By synthesizing the existing evidence from RCTs and cohort studies, we found that comparing with non-bDMARDs, the use of bDMARDs might be associated with reduced risks of MI, heart failure, CV death, all-cause mortality, and 3P-MACE in patients with systemic inflammatory conditions, especially for patients with RA. Moreover, subgroup analyses suggested that the cardioprotective effect of bDMARDs might be prominent in TNF-*α* inhibitor users and patients with follow-up over one year.

This is a state-of-art meta-analysis of biological therapy concerning the risk of CV events in patients with systemic inflammatory diseases. The new insights from this study might give researchers some hints to explore optimal biological therapeutic approaches for better CV prognosis and inspire event-driven clinical trials for CV evaluation of bDMARDs in the future.

Actually, multiple epidemiological studies have implicated the pivotal link between inflammation and cardiovascular events [11, 25–28]. Systemic inflammatory and soluble immune mechanisms (circulating antibodies, immune complexes, and complement activation products) were reported to be involved in accelerating vessel pathology in atherosclerosis [26, 27]. Furthermore, previous studies reported numerous mutual molecular pathways with the pathogenesis of atherosclerosis and persistent inflammation in the development of autoimmune diseases. For example, inflammasome infiltration has been demonstrated to play an indispensable role in the pathological progression of multiple cardiovascular diseases [28]. The formation and activation of inflammatory complex such as NLPR3 inflammasome have been noted in both inflammatory and cardiovascular diseases. And subsequent secretion of interleukin- (IL-) 1 might in turn facilitate the disease development and progression. Moreover, critical proinflammatory cytokines such as IL-6, C-reactive protein (CRP), and tumor necrosis factor (TNF-*α*) not only play a pivotal role in the inflammatory cascade and evolution of rheumatic diseases but also are independent predictors of CVD [11, 29]. Hence, it is plausible that excess CV risk could be observed in systemic inflammatory diseases. Targeting the mutual pathogenic mechanism, intensive anti-inflammatory therapy might have the potent to achieve disease remission and reduce the risk of cardiovascular events in patients with systemic inflammatory conditions. Fortunately, our study has just favorably responded to this scientific hypothesis within such context.

As for disease subtypes, our sensitivity analysis showed that remarkable cardiovascular benefits were observed especially in patients with RA. Consistent with our results, accumulated clinical data favored anti-inflammatory therapies, including bDMARDs, in reducing the cardiovascular risk of RA [8]. However, studies concerning the cardiovascular protective effect of bDMARDs in SLE were limited. Targeting the IFN pathway might yield a promising therapeutic response since IFN was viewed as a major pathogenetic determinant in lupus-related atherosclerosis [30]. In psoriasis, indeed, several studies also suggested a cardioprotective effect of certain biologic agents [16–18, 31]. A recent meta-analysis found that the CV events were less frequent in patients with psoriasis receiving biological therapies targeting IL-17 and IL-23 [32].

Compared with the current available literatures, in addition to patients with RA, we conducted additional analyses in patients with psoriasis and lupus. Unfortunately, we did not see any positive results in these two diseases in our analysis. In our study, it was noted that the sample size of patients with SLE was much less than the patients with RA. One of the possible reasons for the negative results in psoriasis and SLE might be that current sample size and exam power were not enough to validate the cardioprotective effects. Another reason might be associated with the extent of treatment response. Studies on psoriasis suggested that patients with no response to biologic therapy might show minimal reduction in MACE risk [17, 18]. What is more, it was reported that no significant improvement of vascular inflammation was shown in patients with adalimumab treatment [22], and it was also reported that ustekinumab might have the potent to increase the risk of MACE [24]. In addition, the studies on lupus and psoriasis included in this meta-analysis were generally short-term RCTs, and we included few cohort studies related to SLE and psoriasis. While in our analyses, positive results mostly appeared in cohort studies. Therefore, longer follow-up studies are needed to further clarify the long-term effects of CVD.

Several studies demonstrated the associations between TNF-*α* inhibitors and improved CV outcomes, which were consistent with our results. A meta-analysis previously estimated a 30% reduction in risk of cardiovascular events with TNF-*α* inhibitors in RA, with protective effect specifically for MI and stroke [15]. The use of TNF-*α* inhibitor was found to be correlated with improved blood pressure [33], reduced aortic stiffness [34], and improved left ventricular mass index on echocardiography [35]. Possible mechanisms might rely on the quantitative and functional changes in lipids and the improvements in endothelial dysfunction and oxidative stress. It was reported that long-term use of TNF-*α* inhibitor was associated with an increase in HDL, total cholesterol, and triglycerides, while a decrease in apolipoprotein B/A and stable LDL as well as total cholesterol/HDL [36]. Moreover, in RA patients, infliximab treatment led to sustained increases in paraoxonase and arylesterase activities of PON-1 on HDL-cholesterol molecules, which may improve HDL antiatherogenic capacity by increasing the antioxidative properties of HDL [37].

Apart from TNF-*α* inhibitor, CV effects of other bDMARDs were less characterized. A multidatabase cohort study indicated that tocilizumab use had a similar risk of cardiovascular events versus TNF-*α* inhibitor use in patients with RA [19]. Likewise, it was observed that tocilizumab could improve qualitative and functional lipid parameters [38], endothelial function, and oxidative stress [39]. Similarly, improved macro- and microvascular endothelial function and beneficial effects on cholesterol profile were found in small case series of rheumatoid arthritis with rituximab users [20, 21]. Although they may contribute to favorable cardiovascular and metabolic profile, these drugs were not suggested to achieve cardiovascular risk reductions yet in our meta-analysis.

A Danish cohort study, examining the rate of CV events (CV death, MI, and stroke) in 6,902 patients with severe psoriasis, indicated the use of IL-12/23 inhibitor ustekinumab was not associated with reduced CV event rates [23]. Likewise, a meta-analysis of 22 RCTs reported no significant difference in the rate of MACE between anti-IL-12/IL-23 antibody treatments and placebo [18]. Another review found that patients with over-4-year treatment of ustekinumab had a decrease risk in MACE while no effects on MACE were observed with short-term use of ustekinumab [40]. Certainly, with increasing use of non-TNF-*α*-inhibitor bDMARDs, more attention is needed for their cardiovascular profiles.

## 5. Limitations

Our meta-analysis also has several limitations. First, we included both RCTs and cohort studies in our analyses, and subgroup analyses showed that positive results were mainly driven by the result from cohort studies, while we did not observe significant CV risk reduction in RCT strata. Thus, the heterogeneity lying in different study designs and population might compromise the reliability of our results. Therefore, we conducted the sensitivity analyses and metaregression to control the potential bias. But there is no denying that the data should be interpreted with caution, since the unmeasured confounding factors in cohort studies would undermine the confidence of the conclusion. However, since most of included RCTs were not specially designed for cardiovascular evaluation, the synthesized evidence from prospective cohort studies still gave us useful clues for the link between bDMARDs and CV risk reduction. More investigations, such as Cardiovascular Outcome Trials for bDMARDs, should be designed and conducted to validate the aforementioned associations in the future. Second, although we tried our best to collect every available data, the indicators reflecting the alteration of inflammation, such as CRP and ESR, were rarely reported. Furthermore, since the efficacy endpoints varied in different diseases, we were unable to assess the association between disease remission and the risks of CV events uniformly. Moreover, the percentage of participants with history of CVD and its relevant comorbidities was generally low in the included studies. Whether patients with systemic inflammatory conditions who also have established CVD still benefit from bDMARD treatment requires more investigations. Since the included studies were not primarily designed for cardiovascular evaluation, the administration of cardio- and vasculoprotective drugs like angiotensin-converting enzyme inhibitor, *β*-blocker, and anticoagulant was rarely reported, whose influence on patients with bDMARD treatment should be further evaluated in the future.

## 6. Conclusions

According to our meta-analysis, the use of bDMARDs might be associated with reduced risks of CV events in patients with systemic inflammatory conditions, especially for patients with RA. The CV events might be less frequent in TNF-*α* inhibitor users and in bDMARD users with follow-up over one year.

## Figures and Tables

**Table 1:** Sensitivity analyses for the use of bDMARD and incidence of myocardial infarction in patients with systemic inflammatory conditions.

Endpoint	Subgroup		Participant (bDMARD/control)	OR	95% CI	*P* value	*I* ^2^
Myocardial infarction							
	Total		57989/30207	0.74	0.63, 0.87	*0.002*	0
	Study type	Cohort	45040/24148	0.63	0.43, 0.91	*0.01*	66
		RCT	12949/6059	0.79	0.47, 1.31	0.36	0
	Disease type	RA	49510/26524	0.74	0.63, 0.87	*0.0004*	31
		Ps	6324/2675	0.90	0.45, 1.80	0.77	0
		SLE	2155/1008	0.49	0.19, 1.28	0.14	0
	Drug type	TNF-*α* inhibitor	49141/26344	0.74	0.63, 0.88	*0.0006*	27
		IL-17 inhibitor	2358/627	0.31	0.10, 1.00	0.05	3
		IL-23 inhibitor	1046/378	1.25	0.20, 7.94	0.82	0
		IL-12/23 inhibitor	1824/873	1.30	0.33, 5.21	0.71	0
		IL-6 receptor inhibitor	1209/724	1.48	0.35, 6.33	0.60	23
		B cell inhibition	2155/1008	0.49	0.19, 1.28	0.14	0
		T cell inhibition	256/253	0.33	0.01, 8.09	0.50	NA
	Follow-up duration	>1 year	38143/17825	0.73	0.61, 0.87	*0.0006*	37
		<1 year	19846/12382	0.78	0.55, 1.11	0.17	0
	Previous CVD^∗^	<30%	42707/21393	0.78	0.63, 0.96	0.02	34
		30-50%	981/484	3.47	0.18, 67.23	0.41	NA
	Diabetes	<30%	44874/24045	0.61	0.42, 0.88	*0.009*	68
		30-50%	981/484	3.47	0.18, 67.23	0.41	NA
	Hypertension	<30%	29227/11982	0.66	0.39, 1.11	0.12	60
		30-50%	16097/12004	0.77	0.59, 1.01	0.06	0
	Dyslipidemia	<30%	9368/6754	0.27	0.11, 0.62	*0.002*	0
		30-50%	11587/8656	0.77	0.49, 1.20	0.24	NA
		>50%	12568/9140	0.81	0.52, 1.25	0.34	0

^∗^Previous cardiovascular disease includes ischemia heart disease, coronary heart disease, angina, myocardial infarction, congestive heart failure, atrial fibrillation, and cerebrovascular disease. bDMARD: biological disease-modifying antirheumatic drug; CVD: cardiovascular disease; IFN: interferon; IL: interleukin; MACE: major adverse cardiovascular event; NA: not applicable; Ps: psoriasis; RCT: randomized controlled trial; RA: rheumatoid arthritis; SLE: systemic lupus erythematosus; TNF: tumor necrosis factor.

**Table 2:** Sensitivity analyses for the use of bDMARD and incidence of heart failure in patients with systemic inflammatory conditions.

Endpoint	Subgroup		Participant (bDMARD/control)	OR	95% CI	*P* value	*I* ^2^
Heart failure							
	Total		32105/30636	0.84	0.74, 0.95	*0.006*	21
	Study type	Cohort	24511/26848	0.81	0.60, 1.11	0.19	67
		RCT	6725/3248	1.03	0.49, 2.18	0.93	0
	Disease type	RA	28625/28688	0.83	0.73, 0.95	*0.006*	41
		Ps	869/511	0.78	0.14, 4.33	0.78	0
		SLE	2611/1408	0.98	0.40, 2.42	0.96	0
	Drug type	TNF-*α* inhibitor	27786/28274	0.83	0.73, 0.95	*0.005*	43
		IL-23 inhibitor	294/98	1.01	0.04, 24.92	1.00	NA
		IL-6 receptor inhibitor	1158/574	2.49	0.29, 21.35	0.41	0
		B cell inhibition	2233/1126	0.97	0.34, 2.72	0.95	0
		T cell inhibition	454/353	1.00	0.15, 6.61	1.00	0
		IFN receptor antibody	180/182	0.34	0.01, 8.28	0.50	NA
	Follow-up duration	>1 year	18357/20935	0.80	0.69, 0.93	*0.003*	38
		<1 year	13748/9672	0.99	0.75, 1.30	0.93	0
	Previous CVD^∗^	<30%	17575/175/73	0.95	0.77, 1.17	0.62	38
		30-50%	169/88	2.64	0.13, 55.63	0.53	NA
		>50%	225/808	1.20	0.79, 1.84	0.39	NA
	Diabetes	<30%	15647/12092	0.69	0.35, 1.36	0.28	72
		30-50%	1002/4591	1.10	0.81, 1.49	0.53	0
	Hypertension	30-50%	15116/11549	0.83	0.53, 1.28	0.39	59
		>50%	1002/4591	1.10	0.81, 1.49	0.53	0
	Dyslipidemia	>50% (all available studies)	12589/13276	1.04	0.84, 1.28	0.71	0

^∗^Previous cardiovascular disease includes ischemia heart disease, coronary heart disease, angina, myocardial infarction, congestive heart failure, atrial fibrillation, and cerebrovascular disease. bDMARD: biological disease-modifying antirheumatic drug; CVD: cardiovascular disease; IFN: interferon; IL: interleukin; MACE: major adverse cardiovascular event; NA: not applicable; Ps: psoriasis; RCT: randomized controlled trial; RA: rheumatoid arthritis; SLE: systemic lupus erythematosus; TNF: tumor necrosis factor.

**Table 3:** Sensitivity analyses for the use of bDMARD and incidence of cardiovascular death in patients with systemic inflammatory conditions.

Endpoint	Subgroup		Participant (bDMARD/control)	OR	95% CI	*P* value	*I* ^2^
Cardiovascular death							
	Total		15815/10957	0.62	0.40, 0.95	*0.03*	0
	Study type	Cohort	10688/8086	0.40	0.23, 0.69	*0.001*	0
		RCT	5127/2871	1.54	0.69, 3.46	0.29	0
	Disease type	RA	13361/9636	0.60	0.38, 0.96	*0.03*	4
		Ps	2177/1052	0.71	0.18, 2.85	0.63	0
		SLE	277/269	0.97	0.06, 15.60	0.98	NA
	Drug type	TNF-*α* inhibitor	12832/9102	0.53	0.33, 0.86	*0.01*	0
		IL-17 inhibitor	103/22	0.07	0.00, 1.76	0.11	NA
		IL-12/23 inhibitor	1801/894	1.49	0.16, 14.37	0.73	0
		IL-6 receptor inhibitor	288/284	4.97	0.24, 103.88	0.30	NA
		B cell inhibition	277/269	0.97	0.06, 15.60	0.98	NA
		T cell inhibition	514/386	2.17	0.23, 20.91	0.50	0
	Follow-up duration	>1 year	12629/9116	0.46	0.28, 0.77	*0.003*	0
		<1 year	3186/1841	1.62	0.62, 4.20	0.32	0
	Previous CVD^∗^	<30%	9368/6754	0.58	0.25, 1.38	0.22	0
		30-50%	981/484	1.48	0.06, 36.46	0.81	NA
	Diabetes	<30% (all available studies)	10880/7781	0.42	0.21, 0.83	*0.01*	0
	Hypertension	<30%	9368/6754	0.58	0.25, 1.38	0.22	0
		30-50%	981/484	1.48	0.06, 36.46	0.81	NA
	Dyslipidemia	<30%	9368/6754	0.58	0.25, 1.38	0.22	0
		>50%	981/484	1.48	0.06, 36.46	0.81	NA

^∗^Previous cardiovascular disease includes ischemia heart disease, coronary heart disease, angina, myocardial infarction, congestive heart failure, atrial fibrillation, and cerebrovascular disease. bDMARD: biological disease-modifying antirheumatic drug; CVD: cardiovascular disease; IFN: interferon; IL: interleukin; MACE: major adverse cardiovascular event; NA: not applicable; Ps: psoriasis; RCT: randomized controlled trial; RA: rheumatoid arthritis; SLE: systemic lupus erythematosus; TNF: tumor necrosis factor.

**Table 4:** Sensitivity analyses for the use of bDMARD and all-cause mortality in patients with systemic inflammatory conditions.

Endpoint	Subgroup		Participant (bDMARD/control)	OR	95% CI	*P* value	*I* ^2^
All-cause mortality							
	Total		44701/43453	0.64	0.58, 0.70	*<0.0001*	38
	Study type	Cohort	32202/36930	0.60	0.44, 0.82	*0.002*	82
		RCT	12499/6523	0.91	0.61, 1.35	0.65	0
	Disease type	RA	36704/39412	0.64	0.49, 0.85	*0.002*	63
		Ps	36677/1719	0.80	0.26, 2.45	0.70	0
		SLE	4330/2322	0.93	0.54, 1.59	0.79	0
	Drug type	TNF-*α* inhibitor	35879/38727	0.63	0.47, 0.84	*0.002*	66
		IL-6 receptor inhibitor	1159/848	1.36	0.43, 4.31	0.60	0
		IL-17 inhibitor	103/22	0.07	0.00, 1.76	0.11	NA
		IL-23 inhibitor	915/254	0.87	0.09, 8.43	0.91	0
		IL-12/23 inhibitor	1801/894	1.49	0.16, 14.3	0.73	0
		B cell inhibition	3952/2040	1.11	0.59, 2.09	0.74	0
		T cell inhibition	712/486	0.53	0.22, 1.28	0.16	0
		IFN receptor antibody	180/182	3.05	0.12, 75.37	0.50	NA
	Follow-up duration	>1 year	27480/31921	0.62	0.48, 0.80	*0.0002*	42
		<1 year	17221/11532	1.02	0.77, 1.35	0.90	0
	Previous CVD^∗^	<30%	27880/15353	0.81	0.69, 0.95	*0.01*	15
		30-50%	4777/20383	0.58	0.51, 0.67	*<0.0001*	0
		>50%	225/808	0.92	0.56, 1.51	0.75	NA
	Diabetes	<30%	27289/15334	0.68	0.40, 1.14	0.14	80
		30-50%	5002/21191	0.68	0.47, 0.98	*0.04*	0
	Hypertension	<30%	11642/3271	1.19	0.40, 3.55	0.75	NA
		30-50%	16097/12004	0.80	0.68, 0.94	*0.007*	39
		>50%	4021/20707	0.69	0.45, 1.06	0.09	68
	Dyslipidemia	30-50%	3529/2864	0.72	0.59, 0.88	*0.001*	NA
		>50%	116589/29847	0.80	0.53, 1.19	0.26	76

^∗^Previous cardiovascular disease includes ischemia heart disease, coronary heart disease, angina, myocardial infarction, congestive heart failure, atrial fibrillation, and cerebrovascular disease. bDMARD: biological disease-modifying antirheumatic drug; CVD: cardiovascular disease; IFN: interferon; IL: interleukin; MACE: major adverse cardiovascular event; NA: not applicable; Ps: psoriasis; RCT: randomized controlled trial; RA: rheumatoid arthritis; SLE: systemic lupus erythematosus; TNF: tumor necrosis factor.

## Data Availability

All data relevant to the study are included in the article or uploaded as supplementary information. No more additional data are available.

## Additional Points

*Highlights*. (i) This is a comprehensive evaluation regarding the use of bDMARD and CV risk. (ii) The use of bDMARD might be associated with reduced risk of CV events. (iii) The risk reductions were mainly observed in patients with rheumatoid arthritis. (iv) The risk was lower in patients with TNF-*α* inhibitors or follow-up over one year. (v) Such associations need to be validated by well-designed event-driven trials.

## Supplementary Materials

Supplementary Materials Figure S1: funnel plots of analyses for individual and composite CV endpoints. Table S1: baseline characteristics of included randomized controlled trials. Table S2: baseline characteristics of included cohort studies. Table S3: evaluation for risk of bias in included RCTs. Table S4: evaluation for risk of bias in included cohort studies. Table S5: sensitivity analyses for the use of bDMARD and incidence of stroke in patients with systemic inflammatory conditions. Table S6: sensitivity analyses for the use of bDMARD and incidence of composite endpoints in patients with systemic inflammatory conditions. Table S7: metaregression analysis of the association between the use of bDMARDs and the risks of CV events.
