# Supplementary material for: The Biological Disease-Modifying Antirheumatic Drugs and the Risk of Cardiovascular Events: A Systematic Review and Meta-Analysis
Source: Mediators Inflamm. 2021 Aug 31;2021:7712587. doi: 10.1155/2021/7712587 (PMC8423578; doi:10.1155/2021/7712587)
Supplement: Supplementary Materials — Figure S1: funnel plots of analyses for individual and composite CV endpoints. Table S1: baseline characteristics of included randomized controlled trials. Table S2: baseline characteristics of included cohort studies. Table S3: evaluation for risk of bias in included RCTs. Table S4: evaluation for risk of bias in included cohort studies. Table S5: sensitivity analyses for the use of bDMARD and incidence of stroke in patients with systemic inflammatory conditions. Table S6: sensitivity analyses for the use of bDMARD and incidence of composite endpoints in patients with systemic inflammatory conditions. Table S7: metaregression analysis of the association between the use of bDMARDs and the risks of CV events. [file 7712587.f1.docx]

Supplementary Materials

Supplementary Figure legends

Figure S1. Funnel plots of analyses for individual and composite CV endpoints.

Supplementary Table legend

Table S1. Baseline characteristics of included randomized controlled trials.

Table S2. Baseline characteristics of included cohort studies.

Table S3. Evaluation for risk of bias in included RCTs

Table S4. Evaluation for risk of bias in included cohort studies

Table S5. Sensitivity analyses for the use of bDMARD and incidence of stroke in patients with systemic inflammatory conditions

Table S6. Sensitivity analyses for the use of bDMARD and incidence of composite endpoints in patients with systemic inflammatory conditions

Table S7. Meta-regression analysis of the association between the use of bDMARDs and the risks of CV events

Figure S1. Funnel plots of analyses for individual and composite CV endpoints.


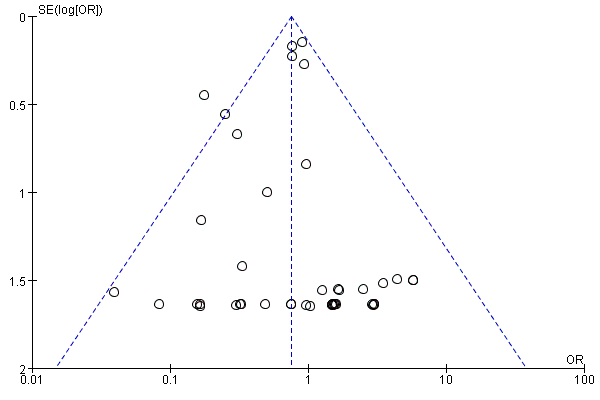

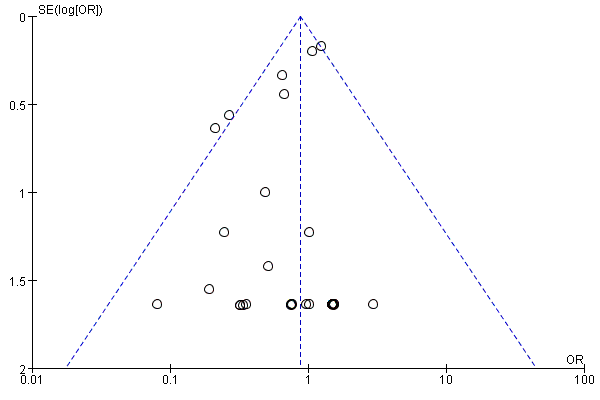

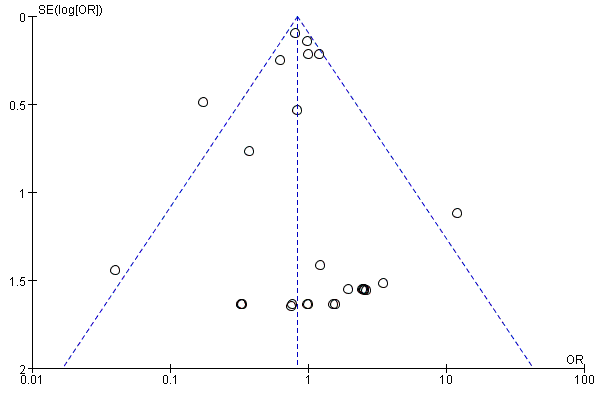


(a) Myocardial infarction (b) Stroke (c) Heart failure


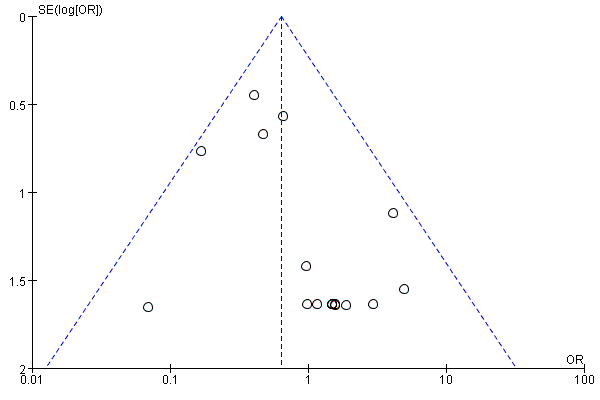

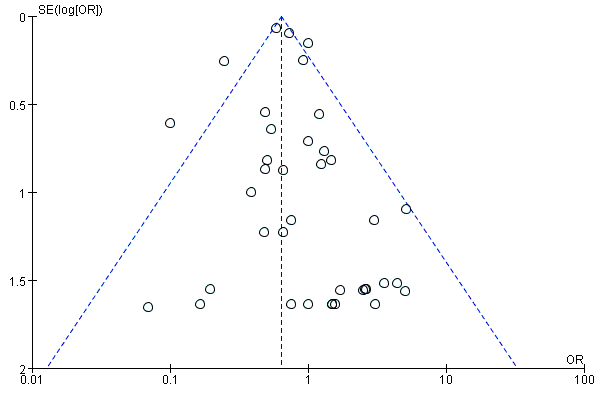

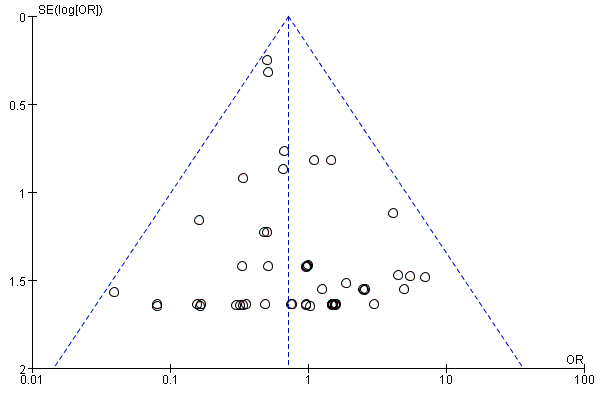


(d) Cardiovascular death (e) All-cause mortality (f) 3P-MACE


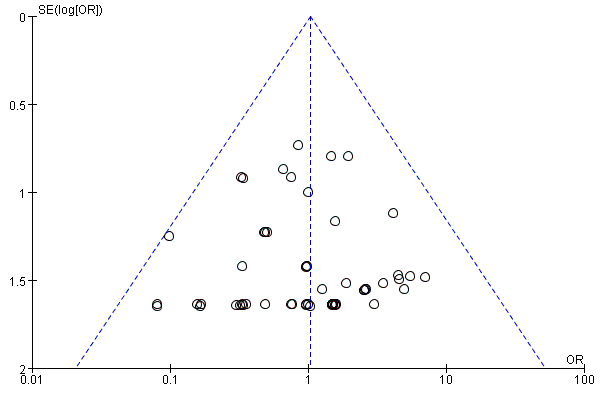


(g) 4P-MACE

Table S1. Baseline characteristics of included randomized controlled trials.

| First author, year | Condition | Study duration | Treatment group | Dosage | No. of patients | Age | Male percentage | Assessed endpoint |
| --- | --- | --- | --- | --- | --- | --- | --- | --- |
| Bejarano 2008^1^ | RA | 56w | ADA |  | 75 | 47 | 58.4 | ACR response, DAS28 remission |
|  |  |  | PBO+MTX |  | 73 | 47 | 53.4 |  |
|  |  |  | PBO |  | 132 | 54.4 | 80.3 |  |
| Bumester 2016^2^ | RA | 52w | TCZ+MTX | 4 mg/kg | 289 | 51.2 | 79.0 | DAS28 remission |
|  |  |  |  | 8 mg/kg | 290 | 49.5 | 79.0 |  |
|  |  |  | TCZ+PBO | 8 mg/kg | 292 | 49.9 | 75.0 |  |
|  |  |  | PBO+MTX |  | 282 | 49.6 | 80.0 |  |
| Detert 2012^3^ | RA | 48w | ADA+MTX | 40mg | 87 | 47.2 | 70.1 | ACR response, DAS28 remission |
|  |  |  | PBO+MTX |  | 85 | 52.5 | 67.1 |  |
| Emery 2008-1^4^ | RA | 24w | TCZ | 8 mg/kg q4w | 175 | 53.9 | 84.0 | ACR response, DAS28 remission |
|  |  |  |  | 4 mg/kg q4w | 163 | 50.9 | 81.0 |  |
|  |  |  | PBO+MTX |  | 160 | 53.4 | 79.0 |  |
| Emery 2008-2^5^ | RA | 52w | ETN+MTX | 50 mg qw | 274 | 50.5 | 74.0 | ACR response, DAS28 remission |
|  |  |  | MTX |  | 268 | 52.3 | 73.0 |  |
| Emery 2017^6^ | RA | 52w | CZP + MTX | 200 mg q2w | 659 | 50.4 | 75.9 | ACR response, DAS28 remission |
|  |  |  | PBO + MTX | 25 mg qw | 217 | 51.2 | 79.8 |  |
| Genovese 2005^7^ | RA | 26w | ABA | 500 /750 /1000 mg | 258 | 53.4 | 77.1 | ACR response, DAS28 remission |
|  |  |  | PBO |  | 133 | 52.7 | 79.7 |  |
| Jones 2010^8^ | RA | 24w | TCZ | 8 mg/kg q4w | 288 | 50.7 | 83.0 | ACR response, DAS28 remission |
|  |  |  | PBO |  | 284 | 50 | 79.0 |  |
| Kavanaugh 2013^9^ | RA | 26w | ADA | 40 mg q2w | 515 | 50.7 | 74.0 | ACR response, DAS28 remission |
|  |  |  | PBO+MTX |  | 517 | 50.4 | 74.0 |  |
| Kay 2008^10^ | RA | 20w | GOL+MTX | 50mg q4w | 37 | 57 | 85.7 | ACR response, DAS28 remission |
|  |  |  |  | 50mg q2w | 32 | 48 | 67.6 |  |
|  |  |  |  | 100mg q4w | 33 | 57.5 | 76.5 |  |
|  |  |  |  | 100mg q2w | 35 | 53.5 | 79.4 |  |
|  |  |  | PBO+MTX |  | 34 | 52.0 | 74.3 |  |
| Kim 2012^11^ | RA | 16w | ETN+MTX | 25 mg w2d | 197 | 48.4 | 91.4 | ACR response, DAS28 remission |
|  |  |  | DMARD+MTX |  | 103 | 48.5 | 88.4 |  |
| Smolen 2008^12^ | RA | 24w | TCZ+MTX | 4mg/kg | 212 | 51.4 | 82.0 | ACR response, DAS28 remission |
|  |  |  |  | 8mg/kg | 206 | 50.8 | 85.0 |  |
|  |  |  | PBO+MTX |  | 204 | 50.6 | 78.0 |  |
| Smolen 2009^13^ | RA | 16w | GOL | 50 mg q4w | 152 | 55 | 74.0 | ACR response, DAS28 remission |
|  |  |  |  | 100 mg q4w | 152 | 55 | 80.0 |  |
|  |  |  | PBO |  | 155 | 54 | 85.0 |  |
| Smolen 2015^14^ | RA | 24w | CTZ | 200mg q2w | 96 | 53.6 | 84.4 | ACR response, DAS28 remission |
|  |  |  | PBO |  | 98 | 54 | 76.5 |  |
| van de Putte 2004^15^ | RA | 26w | ADA | 20 mg q2w | 106 | 53.1 | 79.2 | ACR response |
|  |  |  |  | 20mg qw | 112 | 54.4 | 72.3 |  |
|  |  |  |  | 40mg q2w | 113 | 52.7 | 79.6 |  |
|  |  |  |  | 40mg qw | 103 | 51.8 | 78.6 |  |
|  |  |  | PBO |  | 110 | 53.5 | 77.3 |  |
| Weinblatt 1999^16^ | RA | 24w | ETN+MTX | 25mg | 59 | 48 | 90.0 | ACR response, |
|  |  |  | PBO+MTX |  | 30 | 53.0 | 73.0 |  |
| Weisman 2007^17^ | RA | 16w | ETN | 25 mg w2d | 266 | 60.6 |  |  |
|  |  |  | PBO |  | 269 | 59.3 |  |  |
| Westhovens 2006^18^ | RA | 22w | IFX+MTX | 3mg/kg | 360 | 53 | 80.0 | ACR response, DAS28 remission |
|  |  |  |  | 10mg/kg | 361 | 52 | 77.8 |  |
|  |  |  | PBO+MTX |  | 361 | 52 | 83.2 |  |
| Westhovens 2009^19^ | RA | 52w | ABA+MTX | 10 mg/kg | 256 | 50.1 | 76.6 | ACR response, DAS28 remission |
|  |  |  | PBO+MTX |  | 253 | 49.7 | 78.7 |  |
| St Clair 2004^20^ | RA | 54w | IFX+MTX | 3 mg/kg | 372 | 51 | 71.0 | ACR response, DAS28 remission |
|  |  |  |  | 6 mg/kg | 377 | 50 | 68.0 |  |
|  |  |  | PBO+MTX |  | 291 | 50 | 75.0 |  |
| Yamamoto 2014^21^ | RA | 24w | CTZ+MTX | 100mg q2w | 72 | 54.3 | 80.6 | ACR response, DAS28 remission |
|  |  |  |  | 200mg q2w | 82 | 50.6 | 84.1 |  |
|  |  |  |  | 400mg q2w | 85 | 55.4 | 81.2 |  |
|  |  |  | PBO+MTX |  | 77 | 51.9 | 85.7 |  |
|  |  |  | PBO |  | 107 | 46 | 34.0 |  |
| Blauvelt 2015^22^ | Psoriasis | 12w | SEK | 300mg | 59 | 45.1 | 35.6 | PASI |
|  |  |  |  | 150mg | 59 | 46 | 32.2 |  |
|  |  |  | PBO |  | 59 | 46.5 | 33.9 |  |
| Blauvelt 2017^23^ | Psoriasis | 16w | ADA | 40mg | 333 | 42.9 | 25.4 | PASI |
|  |  |  | GUK | 100mg | 329 | 43.9 | 27.1 |  |
|  |  |  | PBO |  | 174 | 44.9 | 31.6 |  |
| Blauvelt 2020^24^ | Psoriasis | 16w | RIK | 150mg | 407 | 51 | 31.5 | PASI |
|  |  |  | PBO |  | 100 | 48 | 27.0 |  |
| Deodhar 2018^25^ | Psoriasis | 24w | GUK | 100mg | 100 | 47.4 | 48.0 | PASI |
|  |  |  | PBO |  | 49 | 44.2 | 51.0 |  |
| Gottlieb 2009^26^ | Psoriasis | 12w | USK | 90mg*4 | 76 | 50 | 41.0 | PASI |
|  |  |  | PBO |  | 70 | 47.5 | 47.0 |  |
| Gordon 2012^27^ | Psoriasis | 12w | BRK | 200mg | 981 | 45.7 | 32.1 | PASI |
|  |  |  | PBO |  | 484 | 45.1 | 29.1 |  |
| Gordon 2018-1^28^ | Psoriasis | 16w | RIK | 150mg | 304 | 48.3 | 30.0 | PASI |
|  |  |  | USK | 45/90mg | 100 | 46.5 | 30.0 |  |
|  |  |  | PBO |  | 102 | 49.3 | 23.0 |  |
| Gordon 2018-2^28^ | Psoriasis | 16w | RIK | 150mg | 294 | 46.2 | 31.0 | PASI |
|  |  |  | USK | 45/90mg | 99 | 48.6 | 33.0 |  |
|  |  |  | PBO |  | 98 | 46.3 | 32.0 |  |
| Griffiths 2015-1^29^ | Psoriasis | 12w | IXK | 80mg q2w | 350 | 45 | 37.0 | PASI |
|  |  |  |  | 80mg q4w | 347 | 45 | 30.0 |  |
|  |  |  | ETN | 50mg | 357 | 45 | 34.0 |  |
|  |  |  | PBO |  | 167 | 45 | 29.0 |  |
| Griffiths 2015-2^31^ | Psoriasis | 12w | IXK | 80mg q2w | 384 | 46 | 34.0 | PASI |
|  |  |  |  | 80mg q4w | 382 | 46 | 33.0 |  |
|  |  |  | ETN | 50mg | 382 | 46 | 30.0 |  |
|  |  |  | PBO |  | 193 | 46 | 29.0 |  |
| Kavanaugh 2017^30^ | Psoriasis | 24w | GOL | 2mg/kg | 241 | 45.7 | 46.9 | PASI |
|  |  |  | PBO |  | 239 | 46.7 | 49.4 |  |
| Krueger 2007^31^ | Psoriasis | 20w | USK | 45mg | 63 | 46 | 41.0 | PASI |
|  |  |  |  | 90mg | 64 | 46 | 27.0 |  |
|  |  |  |  | 45mg*4 | 63 | 45 | 39.0 |  |
|  |  |  |  | 90mg*4 | 62 | 44 | 19.0 |  |
|  |  |  | PBO |  | 67 | 44 | 28.0 |  |
| Lebwohl 2015-1^32^ | Psoriasis | 12w | BRA | 210mg | 612 | 44.5 | 31.2 | PASI |
|  |  |  |  | 140mg | 607 | 44.8 | 32.3 |  |
|  |  |  | USK | 45/90mg | 300 | 45.4 | 31.7 |  |
|  |  |  | PBO |  | 309 | 43.7 | 29.1 |  |
| Lebwohl 2015-2^32^ | Psoriasis | 12w | BRA | 210mg | 624 | 45.2 | 30.9 | PASI |
|  |  |  |  | 140mg | 629 | 44.6 | 30.5 |  |
|  |  |  | USK | 45/90mg | 313 | 44.8 | 32.3 |  |
|  |  |  | PBO |  | 315 | 44.2 | 34.0 |  |
| Leonardi 2008^33^ | Psoriasis | 12w | USK | 45mg | 255 | 44.8 | 31.4 | PASI |
|  |  |  |  | 90mg | 255 | 46.2 | 32.4 |  |
|  |  |  | PBO |  | 255 | 44.8 | 28.2 |  |
| Mease 2005^34^ | Psoriasis | 24w | ADA | 40mg | 151 | 48.6 | 43.7 | PASI |
|  |  |  | PBO |  | 162 | 49.2 | 45.1 |  |
| Mease 2014^35^ | Psoriasis | 24w | CZP | 200mg q2w | 138 | 48.2 | 53.6 | PASI |
|  |  |  |  | 400mg q4w | 135 | 47.1 | 54.1 |  |
|  |  |  | PBO |  | 136 | 47.3 | 58.1 |  |
| Mease 2015^36^ | Psoriasis | 16w | SEK | 150mg | 202 | 49.6 | 52.5 | PASI |
|  |  |  |  | 75mg | 202 | 48.8 | 58.4 |  |
|  |  |  | PBO |  | 202 | 48.5 | 52.5 |  |
| Mease 2020^37^ | Psoriasis | 24w | GUK | 100mg q4w | 245 | 45.9 | 42.0 | PASI |
|  |  |  |  | 100mg q8w | 248 | 44.9 | 48.0 |  |
|  |  |  | PBO |  | 246 | 46.3 | 52.0 |  |
| Nakagawa 2016^38^ | Psoriasis | 12w | BRA | 70mg | 39 | 43.4 | 12.8 | PASI |
|  |  |  |  | 140mg | 37 | 46.4 | 18.9 |  |
|  |  |  |  | 210mg | 37 | 46.4 | 21.6 |  |
|  |  |  | PBO |  | 38 | 46.6 | 28.9 |  |
| Papp 2008^39^ | Psoriasis | 12w | USK | 45mg | 409 | 45.1 | 30.8 | PASI |
|  |  |  |  | 90mg | 411 | 46.6 | 33.3 |  |
|  |  |  | PBO |  | 410 | 47 | 31.0 |  |
| Papp 2013^40^ | Psoriasis | 36w | SEK | 25mg*1 | 29 | 46.1 | 31.0 | PASI |
|  |  |  |  | 25mg*3 | 26 | 46.3 | 15.4 |  |
|  |  |  |  | 75mg*3 | 21 | 45.8 | 33.3 |  |
|  |  |  |  | 150mg*3 | 27 | 45.4 | 22.2 |  |
|  |  |  | PBO |  | 22 | 45.9 | 36.4 |  |
| Reich 2017^41^ | Psoriasis | 16w | ADA | 40mg | 248 | 43.2 | 31.5 | PASI |
|  |  |  | GUK | 100mg | 494 | 43.7 | 29.6 |  |
|  |  |  | PBO |  | 248 | 43.3 | 30.2 |  |
| Reich 2017 reSURFACE 1^42^ | Psoriasis | 12w | TIK | 200mg | 308 | 46.9 | 27.0 | PASI |
|  |  |  |  | 100mg | 309 | 46.4 | 33.0 |  |
|  |  |  | PBO |  | 154 | 47.9 | 35.0 |  |
| Furie 2011^43^ | SLE | 76w | BEM+PRED | 1mg/kg | 271 | 40 | 93.4 | SRI Response Rate ≥4, SLEDAI |
|  |  |  |  | 10mg/kg | 273 | 40.5 | 94.9 |  |
|  |  |  | PBO+PRED |  | 275 | 40 | 91.6 |  |
| Furie 2014-1^44^ | SLE | 52w | ABA+PRED | 30/10mg/kg | 99 | 31 | 84.8 | MCR, PCR |
|  |  |  |  | 10/10mg/kg | 99 | 30.5 | 86.9 |  |
|  |  |  | PBO+PRED |  | 100 | 31.8 | 81.0 |  |
| Furie 2014-2^45^ | SLE | 52w | BLD+PRED | 100mg qw | 93 | 35.8 | 91.4 | SRI Response Rate ≥4, SLEDAI |
|  |  |  |  | 200mg qw | 92 | 38.4 | 93.5 |  |
|  |  |  |  | 200mg q4w | 92 | 37 | 92.4 |  |
|  |  |  | PBO+PRED |  | 269 | 37.9 | 95.5 |  |
| Ginzler 2014^46^ | SLE | 52w | BEM+PRED | 1mg/kg | 114 | 42 | 93.9 | SRI Response Rate ≥4, BILAG, SLEDAI |
|  |  |  |  | 4mg/kg | 111 | 42.6 | 94.6 |  |
|  |  |  |  | 10mg/kg | 111 | 41.8 | 94.6 |  |
|  |  |  | PBO+PRED |  | 113 | 42.2 | 90.3 |  |
| Isenberg 2015^47^ | SLE | 76w | ATA+PRED | 75mg qw | 157 | 39.1 | 93.1 |  |
|  |  |  |  | 150mg qw | 144 | 39 | 92.4 |  |
|  |  |  | PBO+PRED |  | 154 | 39 | 94.3 |  |
| Isenberg 2016^48^ | SLE | 52w | TAB+PRED | 120mg q2w | 386 | 40 | 92.9 | SRI Response Rate ≥4, SLEDAI |
|  |  |  |  | 120mg q4w | 389 | 40 | 93.1 |  |
|  |  |  | PBO+PRED |  | 387 | 39 | 95.0 |  |
| Khamashta 201649 | SLE | 74w | SFA+PRED | 200mg | 108 | 39.9 | 95.4 | SRI Response Rate ≥4, BILAG, SLEDAI |
|  |  |  |  | 600mg | 108 | 40 | 89.8 |  |
|  |  |  |  | 1200mg | 107 | 39.4 | 90.7 |  |
|  |  |  | PBO+PRED |  | 108 | 38.4 | 93.5 |  |
| Merrill 2011^50^ | SLE | 96w | RIT+PRED  PBO+PRED | 1000mg | 169 | 40.2 | 89.9 | MCR, MCR+PCR, PCR, BILAG, |
|  |  |  |  |  | 88 | 40.5 | 93.2 |  |
| Merrill 2016^51^ | SLE | 52w | TAB+PRED | 120mg q2w | 371 | 42 | 91.9 | SRI Response Rate ≥4, SLEDAI |
|  |  |  |  | 120mg q4w | 374 | 41 | 92.0 |  |
|  |  |  | PBO+PRED |  | 376 | 42 | 92.8 |  |
| Morand 2020^52^ | SLE | 52w | ANF+PRED | 300mg | 180 | 43.1 | 93.3 | SLEDAI |
|  |  |  | PBO+PRED |  | 182 | 41.1 | 93.4 |  |
| Navarra 2011^53^ | SLE | 52w | BEM+PRED | 1mg/kg | 288 | 35 | 94.1 | SRI Response Rate ≥4 |
|  |  |  |  | 10mg/kg | 290 | 35.4 | 96.6 |  |
|  |  |  | PBO+PRED |  | 287 | 36.2 | 94.1 |  |
| Rovin 2012^54^ | SLE | 78w | RIT+PRED | 1000 mg | 73 | 31.8 | 87.5 | MCR, MCR+PCR, PCR, BILAG, |
|  |  |  | PBO+PRED |  | 71 | 29.4 | 93.1 |  |
| Wallace 2014^55^ | SLE | 12w | EPA+PRED | 100mg q2w | 39 | 41 | 92.3 | BILAG, SLEDAI |
|  |  |  |  | 400mg q2w | 37 | 38.6 | 92.1 |  |
|  |  |  |  | 600mg q2w | 35 | 37.2 | 100.0 |  |
|  |  |  |  | 1200mg q2w | 37 | 37.2 | 100.0 |  |
|  |  |  |  | 1800mg q2w | 39 | 38 | 92.1 |  |
|  |  |  | PBO+PRED |  | 38 | 41.1 | 86.8 |  |

*ABA, abatacept; ACR response, American College of Rheumatology improvement criteria; ADA, adalimumab; ANF, Anifrolumab; ATA, atacicept; BEM, belimumab; BILAG, British Isles Lupus Assessment Group; BLD, blisibimod; BRA, brodalumab; BRK, briakinumab; CZP, certolizumab pegol; DAS28, Disease Activity Score in 28 joints; ETN, etanercept; EPA, epratuzumab;GOL, golimumab; GUK, guselkumab; IFX, Infliximab; IXK, ixekizumab; MTX, methotrexate; MCR, major clinical response; PASI, psoriasis area and severity index; PBO, placebo; PCR, partial clinical response; PRED, prednisone; RA, rheumatoid arthritis; RIK, risankizumab; RIT, rituximab; SEK, secukinumab; SFA, sifalimumab; SLE, systemic lupus erythematosus; SLEDAI, SLE Disease Activity Index; SRI, SLE Responder Index; TAB, tabalumab; TCZ, tocilizumab; TIK, tildrakizumab; USK, ustekinumab*

Table S2. Baseline characteristics of included cohort studies.

| First author, year | Condition | Cardiovascular endpoint | Study duration | Treatment group | No. of patients | Age | Female percentage |
| --- | --- | --- | --- | --- | --- | --- | --- |
| AL-ALY 2011^56^ | RA | Atherosclerotic heart disease, congestive heart failure, peripheral artery disease, or cerebrovascular disease, and on the risk of death | 3.5y | TNF-α inhibitor | 3796 | 57 | 9.0 |
|  |  |  |  | Other DMARD | 19899 | 63 | 9.0 |
| Bili 2014^57^ | RA | Myocardial infarction, unstable angina, or coronary revascularization procedure | 3.4y | TNF-α inhibitor | 1022 | 51.7 | 71.7 |
|  |  |  |  | MTX | 1698 | 56.2 | 72.8 |
|  |  |  |  | Non-methotrexate non-biological DMARDs | 1131 | 56.9 | 74.8 |
| Carmona 2007^58^ | RA | Ischemic heart disease, cardiac failure and stroke | 5y | TNF-α inhibitor | 789 | 59 | 79.0 |
|  |  |  |  | No bDMARDs | 789 | 61 | 72.0 |
| Curtis 2007^59^ | RA | Heart failure | 1.5y | IFX | 330 | 40 | 70.0 |
|  |  |  |  | ETN | 808 | 38 | 75.0 |
|  |  |  |  | No anti-TNF | 983 | 39 | 75.0 |
| Dixon 2007^60^ | RA | Myocardial infarction | 1.66y | TNF-α inhibitor | 8659 | 56 | 76.0 |
|  |  |  |  | DMARD | 2170 | 60 | 72.0 |
| Geborek 2002^61^ | RA | Cardiovascular events | 1y | ETN | 166 | 54 | 78.0 |
|  |  |  |  | IFX | 135 | 55.4 | 79.0 |
|  |  |  |  | Leflunomide | 103 | 61.3 | 82.0 |
| Greenberg 2010^62^ | RA | Myocardial infarction, transient ischemic attack, stroke and cardiovascular death | 1.9y | TNF-α inhibitor | 4684 | 56.3 | 76.9 |
|  |  |  |  | MTX | 4969 | 59.09 | 74.2 |
|  |  |  |  | Non-methotrexate nbDMARDs | 1785 | 58.6 | 75.3 |
| Jacobsson 2005^63^ | RA | Cardiovascular events | 1y | TNF-α inhibitor | 531 | 55 | 78.0 |
|  |  |  |  | No anti-TNF | 543 | 61 | 75.0 |
| Cole 2007^64^ | RA | Heart failure | NA | TNF-α inhibitor | 103 | 58.7 | 10.7 |
|  |  |  |  | No anti-TNF | 100 | 67.6 | 7.0 |
| Ljung 2012^65^ | RA | Acute coronary syndromes | 3.9y | TNF-α inhibitor | 1271 | 50.5 | 74.7 |
|  |  |  |  | No anti-TNF | 4729 | 58.5 | 69.3 |
| Low 2016^66^ | RA | Ischemic Stroke | 5.6y | TNF-α inhibitor | 11642 | 56 | 76.5 |
|  |  |  |  | sDMARDs | 3271 | 59.9 | 73.5 |
| Low 2017^67^ | RA | Myocardial infarction | 5.3y | TNF-α inhibitor | 11200 | 55.6 | 78.0 |
|  |  |  |  | sDMARDs | 3058 | 59.5 | 75.0 |
| Morgan 2014^68^ | RA | Cardiovascular events or all-cause mortality | 4.8y | ETN | 3529 | 55.3 | 77.3 |
|  |  |  |  | Conventional DMARDs | 2864 | 59.8 | 74.5 |
| Setoguchi 2008^69^ | RA | Heart failure | With HF: 1.6y | TNF-α inhibitor | 225 | 73 | 89.0 |
|  |  |  |  | MTX | 808 | 77 | 84.0 |
|  |  |  | Without HF: 1.8y | TNF-α inhibitor | 777 | 72 | 90.0 |
|  |  |  |  | MTX | 3783 | 74 | 89.0 |
| Solomon 2012^70^ | RA | Heart failure | NA | TNF-α inhibitor | 11587 | 55.4 | 86.5 |
|  |  |  |  | nbDMARD | 8656 | 56.2 | 85.9 |
| Solomon 2013^71^ | RA | Myocardial infarction, stroke or coronary re-vascularization | 0.5y | TNF-α inhibitor | 11587 | 55.4 | 86.5 |
|  |  |  |  | nbDMARD | 8656 | 56.2 | 85.9 |
| Wolfe 2004^72^ | RA | Heart failure | NA | TNF-α inhibitor | 5832 | 60 | 78.0 |
|  |  |  |  | IFX | 4152 | 61.5 | 77.0 |
|  |  |  |  | ETN | 1525 | 56.7 | 80.0 |
|  |  |  |  | No anti-TNF | 7339 | 61.5 | 76.0 |

*TNF, tumor necrosis factor; DMARD, disease-modifying antirheumatic drug; ETN, etanercept; IFX, Infliximab; MTX, methotrexate; nbDMARD, non-biological DMARD; RA, rheumatoid arthritis; sDMARD, synthetic DMARD.*

Table S3. Evaluation for risk of bias in included RCTs

| Study | Selection bias (random sequence generation) | Selection bias (allocation concealment) | Performance bias | Detection bias | Attrition bias | Reporting bias | Other bias |
| --- | --- | --- | --- | --- | --- | --- | --- |
| Bejarano 2008^1^ | Low risk | Low risk | Low risk | Low risk | High risk | Low risk | Low risk |
| Bumester 2016^2^ | Low risk | Low risk | Low risk | Low risk | Low risk | Low risk | Low risk |
| Detert 2012^3^ | Low risk | Low risk | Low risk | Low risk | Low risk | Low risk | Low risk |
| Emery 2008-1^4^ | Low risk | Low risk | Low risk | Low risk | Low risk | Low risk | Low risk |
| Emery 2008-2^5^ | Low risk | Low risk | Low risk | Low risk | Low risk | Low risk | Low risk |
| Emery 2017^6^ | Low risk | Low risk | Low risk | Low risk | High risk | Low risk | Low risk |
| Genovese 2005^7^ | Low risk | Low risk | Low risk | Low risk | Low risk | Low risk | Low risk |
| Jones 2010^8^ | Low risk | Low risk | Low risk | Low risk | Low risk | Low risk | Low risk |
| Kavanaugh 2013^9^ | Low risk | Unclear risk | Low risk | Low risk | Low risk | Low risk | Low risk |
| Kay 2008^10^ | Low risk | Low risk | Low risk | Low risk | High risk | Low risk | Low risk |
| Kim 2012^11^ | Low risk | High risk | High risk | Low risk | High risk | Low risk | Low risk |
| Smolen 2008^12^ | Low risk | Unclear risk | Low risk | Low risk | Low risk | Low risk | Low risk |
| Smolen 2009^13^ | Low risk | Low risk | Low risk | Low risk | Low risk | Low risk | Low risk |
| Smolen 2015^14^ | Low risk | Low risk | High risk | Low risk | Low risk | Low risk | Low risk |
| van de Putte 2004^15^ | Low risk | Unclear risk | Low risk | Low risk | High risk | Low risk | Low risk |
| Weinblatt 1999^16^ | Low risk | Unclear risk | Low risk | Low risk | Unclear risk | Low risk | Low risk |
| Weisman 2007^17^ | Unclear risk | Unclear risk | Low risk | Low risk | Unclear risk | Low risk | Low risk |
| Westhovens 2006^18^ | Unclear risk | Unclear risk | Low risk | Low risk | Low risk | Low risk | Low risk |
| Westhovens 2009^19^ | Unclear risk | Unclear risk | Low risk | Low risk | Low risk | Low risk | Low risk |
| St Clair^20^ | Low risk | Low risk | Unclear risk | Low risk | Low risk | Low risk | Low risk |
| Yamamoto 2014^21^ | Low risk | Low risk | Low risk | Low risk | High risk | Low risk | Low risk |
| Blauvelt 2015^22^ | Low risk | Low risk | Low risk | Low risk | Low risk | Low risk | Low risk |
| Blauvelt 2017^23^ | Low risk | Low risk | Low risk | Low risk | Low risk | Low risk | Low risk |
| Blauvelt 2020^24^ | Low risk | Low risk | Low risk | Low risk | Low risk | Low risk | Low risk |
| Deodhar 2018^25^ | Low risk | Low risk | Low risk | Low risk | Low risk | Low risk | Low risk |
| Gottlieb 2009^26^ | Low risk | Low risk | Low risk | Low risk | Low risk | Low risk | Low risk |
| Gordon 2012^27^ | Low risk | Low risk | Low risk | Low risk | High risk | Low risk | Low risk |
| Gordon 2018^28^ | Low risk | Low risk | Low risk | Low risk | Low risk | Low risk | Low risk |
| Griffiths 2015^29^ | Unclear risk | Low risk | Low risk | Low risk | Low risk | Low risk | Low risk |
| Kavanaugh 2017^30^ | Low risk | Low risk | Low risk | Low risk | Low risk | Low risk | Low risk |
| Krueger 2007^31^ | Low risk | Low risk | Low risk | Low risk | High risk | Low risk | Low risk |
| Lebwohl 2015^32^ | Low risk | Low risk | Low risk | Low risk | Low risk | Low risk | Low risk |
| Leonardi 2008^33^ | Low risk | Low risk | Low risk | Low risk | Low risk | Low risk | Low risk |
| Mease 2005^34^ | Unclear risk | Low risk | Low risk | Low risk | Low risk | Low risk | Low risk |
| Mease 2014^35^ | Unclear risk | Low risk | Low risk | Low risk | Low risk | Low risk | Low risk |
| Mease 2015^36^ | Low risk | Low risk | Low risk | Low risk | Low risk | Low risk | Low risk |
| Mease 2020^37^ | Low risk | Low risk | Low risk | Low risk | Low risk | Low risk | Low risk |
| Nakagawa 2016^38^ | Unclear risk | Low risk | Low risk | Low risk | Low risk | Low risk | Low risk |
| Papp 2008^39^ | Low risk | Low risk | Low risk | Low risk | Low risk | Low risk | Low risk |
| Papp 2013^40^ | Low risk | Low risk | Low risk | Low risk | High risk | Low risk | Low risk |
| Reich 2017^41^ | Low risk | Low risk | Low risk | Low risk | Low risk | Low risk | Low risk |
| Reich 2017 reSURFACE 1 ^42^ | Low risk | Low risk | Low risk | Low risk | Low risk | Low risk | Low risk |
| Furie 2011^43^ | Low risk | Low risk | Low risk | Low risk | High risk | Low risk | Low risk |
| Furie 2014-1^44^ | Unclear risk | Low risk | Low risk | Low risk | High risk | Low risk | Low risk |
| Furie 2014-2^45^ | Low risk | Low risk | Low risk | Low risk | High risk | Low risk | Low risk |
| Ginzler 2014^46^ | Unclear risk | Low risk | Low risk | Low risk | Low risk | Low risk | Low risk |
| Isenberg 2015^47^ | Unclear risk | Low risk | Low risk | Low risk | High risk | Low risk | Low risk |
| Isenberg 2016^48^ | Unclear risk | Low risk | Low risk | Low risk | High risk | Low risk | Low risk |
| Khamashta 2016^49^ | Low risk | Low risk | Low risk | Low risk | Low risk | Low risk | Low risk |
| Merrill 2011^50^ | Unclear risk | Low risk | Low risk | Low risk | High risk | Low risk | Low risk |
| Merrill 2016^51^ | Unclear risk | Low risk | Low risk | Low risk | High risk | Low risk | Low risk |
| Morand 2020^52^ | Low risk | Low risk | Low risk | Low risk | High risk | Low risk | Low risk |
| Navarra 2011^53^ | Low risk | Low risk | Low risk | Low risk | High risk | Low risk | Low risk |
| Rovin 2012^54^ | Unclear risk | Low risk | Low risk | Low risk | Low risk | Low risk | Low risk |
| Wallace 2014^55^ | Low risk | Low risk | Low risk | Low risk | High risk | Low risk | Low risk |

Table S4. Evaluation for risk of bias in included cohort studies

| Studies | NOS Evaluation | | | | | | | |
| --- | --- | --- | --- | --- | --- | --- | --- | --- |
|  | 1 | 2 | 3 | 4 | 5 | 6 | 7 | 8 |
| AL-ALY 2011^56^ | a | a | b | b | b | a | a | b |
| Bili 2014^57^ | a | a | a | b | b | a | a | b |
| Carmona 2007^58^ | a | b | a | b | b | a | a | a |
| Curtis 2007^59^ | b | a | a | b | a | a | a | b |
| Dixon 2007^60^ | a | a | a | b | a | b | a | b |
| Geborek 2002^61^ | a | a | a | a | a | c | a | b |
| Greenberg 2010^62^ | a | a | a | b | b | a | a | b |
| Jacobsson 2005^63^ | a | b | a | b | b | b | a | b |
| Cole 2007^64^ | a | a | a | b | b | a | b | d |
| Ljung 2012^65^ | a | a | a | b | b | a | a | b |
| Low 2016^66^ | a | a | a | b | b | a | a | b |
| Low 2017^67^ | a | a | a | b | b | b | a | b |
| Morgan 2014^68^ | a | a | a | a | b | b | a | b |
| Setoguchi 2008^69^ | a | a | a | b | b | a | a | b |
| Solomon 2012^70^ | a | a | a | b | b | a | a | d |
| Solomon 2013^71^ | a | a | a | b | b | a | a | b |
| Wolfe 2004^72^ | a | a | a | b | a | c | a | d |

NOS quality assessment scale for case cohort studies: 1. Representativeness of the exposed cohort: a. Truly representative of the average in the community; b. Somewhat representative of the average in the community; c. Selected group of users e.g. nurses, volunteers; d. No description of the derivation of the cohort. 2. Selection of the non-exposed cohort: a. Drawn from the same community as the exposed cohort; b. Drawn from a different source; c. No description of the derivation of the non-exposed cohort. 3. Ascertainment of exposure: a. Secure record (e.g. surgical records); b. Structured interview; c. Written self-report; d. No description. 4. Demonstration that outcome of interest was not present at start of study: a. Yes; b. No. 5. Comparability of cohorts on the basis of the design or analysis: a. Study controls for (Select the most important factor); b. Study controls for any additional factor (These criteria could be modified to indicate specific control for a second important factor.). 6. Assessment of outcome: a. Independent blind assessment; b. Record linkage; c. Self-report; d. No description. 7. Was follow-up long enough for outcomes to occur: a. Yes (select an adequate follow-up period for outcome of interest); b. No. 8. Adequacy of follow up of cohorts: a. Complete follow up – all subjects accounted for; b. Subjects lost to follow up unlikely to introduce bias – small number lost; c. Follow up rate is low and no description of those lost; d. No statement.

Table S5. Sensitivity analyses for the use of bDMARD and incidence of stroke in patients with systemic inflammatory conditions

| End point | | Subgroup |  | Participant (bDMARD/control) | OR | 95 % CI | P value | I^2^ |
| --- | --- | --- | --- | --- | --- | --- | --- | --- |
|  | Stroke | | | | | | | |
|  | | Total |  | 44439/24662 | 0.88 | 0.72,1.07 | 0.21 | 0 |
|  | | Study type | Cohort | 33917/20013 | 0.70 | 0.44,1.12 | 0.14 | 67 |
|  | |  | RCT | 10522/4649 | 0.67 | 0.36,1.23 | 0.19 | 0 |
|  | | Disease type | RA | 35549/21183 | 0.90 | 0.73,1.11 | 0.31 | 39 |
|  | |  | Ps | 6902/2496 | 0.80 | 0.34,1.88 | 0.60 | 0 |
|  | |  | SLE | 1988/983 | 0.60 | 0.20,1.75 | 0.35 | 0 |
|  | | Drug type | TNF-α inhibitor | 35164/20888 | 0.91 | 0.74,1.12 | 0.37 | 36 |
|  | |  | IL-17 inhibitor | 3768/1078 | 1.03 | 0.24,4.30 | 0.97 | 0 |
|  | |  | IL-23 inhibitor | 900/346 | 0.39 | 0.06,2.57 | 0.33 | 37 |
|  | |  | IL-12/23 inhibitor | 1748/803 | 1.22 | 0.19,7.75 | 0.83 | 0 |
|  | |  | IL-6 receptor inhibitor | 871/564 | 0.34 | 0.07,1.70 | 0.19 | 0 |
|  | |  | B cell inhibition | 1988/983 | 0.60 | 0.20,1.75 | 0.35 | 0 |
|  | | Follow-up duration | >1 year | 25463/13172 | 0.83 | 0.65,1.06 | 0.13 | 29 |
|  | |  | <1 year | 18976/11490 | 0.99 | 0.70,1.42 | 0.97 | 0 |
|  | | Previous CVD | <30% | 33368/18874 | 1.05 | 0.83,1.32 | 0.69 | 3 |
|  | |  | 30-50% | 981/484 | 1.48 | 0.06,36.46 | 0.81 | NA |
|  | | Diabetes | <30% (All available studies) | 34880/19901 | 0.98 | 0.78,1.22 | 0.84 | 38 |
|  | | Hypertension | <30% | 22312/10761 | 0.72 | 0.41,1.26 | 0.24 | 57 |
|  | |  | 30-50% | 981/484 | 1.48 | 0.06,36.46 | 0.81 | NA |
|  | | Dyslipidemia | <30% | 10139/6947 | 0.65 | 0.39,1.10 | 0.11 | 0 |
|  | |  | >50% | 12568/9140 | 1.08 | 0.73,1.61 | 0.70 | 0 |

Table S6. Sensitivity analyses for the use of bDMARD and incidence of composite endpoints in patients with systemic inflammatory conditions

| End point | | Subgroup |  | Participant (bDMARD/control) | OR | 95 % CI | P value | I^2^ |
| --- | --- | --- | --- | --- | --- | --- | --- | --- |
|  | 3P-MACE | | | | | | | |
|  | | Total |  | 28611/16185 | 0.69 | 0.53,0.89 | **0.004** | 0 |
|  | | Study type | Cohort | 9368/6754 | 0.51 | 0.34,0.75 | **0.0007** | 0 |
|  | |  | RCT | 19243/9431 | 0.87 | 0.62,1.22 | 0.42 | 0 |
|  | | Disease type | RA | 15727/10653 | 0.64 | 0.47,0.88 | **0.005** | 0 |
|  | |  | Ps | 10283/4167 | 0.91 | 0.51,1.59 | 0.73 | 0 |
|  | |  | SLE | 2601/1365 | 0.63 | 0.31,1.31 | 0.22 | 0 |
|  | | Drug type | TNF-α inhibitor | 15438/10279 | 0.61 | 0.45,0.84 | **0.003** | 0 |
|  | |  | IL-17 inhibitor | 3976/1138 | 0.64 | 0.24,1.69 | 0.37 | 0 |
|  | |  | IL-23 inhibitor | 1946/723 | 0.74 | 0.21,2.65 | 0.64 | 0 |
|  | |  | IL-12/23 inhibitor | 2639/1286 | 1.75 | 0.51,6.00 | 0.37 | 0 |
|  | |  | IL-6 receptor inhibitor | 1497/1008 | 1.06 | 0.38,2.95 | 0.91 | 0 |
|  | |  | B cell inhibition | 2601/1365 | 0.63 | 0.31,1.31 | 0.22 | 0 |
|  | |  | T cell inhibition | 514/386 | 1.21 | 0.15,9.62 | 0.86 | 0 |
|  | | Follow-up duration | >1 year | 14823/9712 | 0.59 | 0.43,0.80 | **0.0008** | 0 |
|  | |  | <1 year | 13788/6473 | 0.95 | 0.61,1.47 | 0.81 | 0 |
|  | | Previous CVD | <30% | 11371/7696 | 0.49 | 0.34,0.71 | **0.0002** | 0 |
|  | |  | 30-50% | 1150/572 | 2.18 | 0.37,13.00 | 0.39 | 29 |
|  | | Diabetes | <30% | 12619/8186 | 0.53 | 0.37,0.76 | 0.0005 | 0 |
|  | |  | 30-50% | 169/88 | 0.52 | 0.03,8.38 | 0.64 | NA |
|  | | Hypertension | <30% | 10516/7140 | 0.50 | 0.34/0.73 | **0.0004** | 0 |
|  | |  | 30-50% | 981/484 | 5.46 | 0.30,98.90 | 0.25 | NA |
|  | | Dyslipidemia | <30% | 10516/7140 | 0.50 | 0.34,0.73 | **0.0004** | 0 |
|  | |  | >50% | 981/484 | 5.46 | 0.30,98.90 | 0.25 | NA |
|  | 4P-MACE | | | | | | | |
|  | | Total (all RCTs) |  | 21435/10574 | 0.99 | 0.72,1.35 | 0.94 | 0 |
|  | | Disease type | RA | 6359/3899 | 1.08 | 0.65,1.78 | 0.77 | 0 |
|  | |  | Ps | 10577/4265 | 0.97 | 0.56,1.70 | 0.92 | 0 |
|  | |  | SLE | 4499/2410 | 0.88 | 0.49,1.60 | 0.68 | 0 |
|  | | Drug type | TNF-α inhibitor | 6070/3525 | 1.04 | 0.61,1.79 | 0.89 | 0 |
|  | |  | IL-17 inhibitor | 3976/1138 | 0.72 | 0.27,1.95 | 0.52 | 0 |
|  | |  | IL-23 inhibitor | 2240/821 | 0.77 | 0.24,2.52 | 0.67 | 0 |
|  | |  | IL-12/23 inhibitor | 2639/1286 | 1.75 | 0.51,6.00 | 0.37 | 0 |
|  | |  | IL-6 receptor inhibitor | 1497/1008 | 1.24 | 0.46,3.36 | 0.67 | 10 |
|  | |  | B cell inhibition | 4121/2128 | 0.87 | 0.47,1.62 | 0.66 | 0 |
|  | |  | T cell inhibition | 712/486 | 1.11 | 0.25,4.93 | 0.89 | 0 |
|  | |  | IFN receptor antibody | 180/182 | 0.34 | 0.01,8.28 | 0.50 | NA |
|  | | Follow-up duration | >1 year | 7353/4003 | 0.95 | 0.60,1.51 | 0.82 | 0 |
|  | |  | <1 year | 14082/6571 | 1.02 | 0.66,1.57 | 0.92 | 0 |
|  | | Previous CVD | <30% | 2270/948 | 0.50 | 0.20,1.28 | 0.15 | 0 |
|  | |  | 30-50% | 1150/572 | 2.89 | 0.51,16.57 | 0.23 | 0 |
|  | | Diabetes | <30% (All available studies) | 2129870 | 1.12 | 0.30,4.21 | 0.86 | 32 |
|  | | Hypertension | <30% | 1148/386 | 0.32 | 0.05,1.97 | 0.22 | 0 |
|  | |  | 30-50% | 981/484 | 5.46 | 0.30,98.90 | 0.25 | NA |
|  | | Dyslipidemia | <30% | 1148/386 | 0.32 | 0.05,1.97 | 0.22 | 0 |
|  | |  | >50% | 981/484 | 5.46 | 0.30,98.90 | 0.25 | NA |

Table S7. Meta-regression analysis of the association between the use of bDMARDs and the risks of CV events

| Disease/Endpoint | Parameter | Coefficient | 95 % CI | P value |
| --- | --- | --- | --- | --- |
| RA RCT |  |  |  |  |
| MI |  |  |  |  |
|  | Age | 0.302 | -0.26,0.863 | 0.262 |
|  | Female percentage | -0.457 | -21.868,20.954 | 0.963 |
|  | Study duration | 0.048 | -0.007,0.102 | 0.079 |
|  | Baseline CRP | 0.308 | -0.817,1.433 | 0.556 |
|  | CRP change | 1.75 | -3.557,7.057 | 0.292 |
|  | Baseline ESR | -0.063 | -0.367,0.241 | 0.615 |
|  | ACR 20 | -1.219 | -9.959,7.521 | 0.751 |
|  | ACR 50 | -1.037 | -8352,6.277 | 0.752 |
|  | ACR 70 | -0.582 | -8.182,7.018 | 0.864 |
| Stroke |  |  |  |  |
|  | Age | 0.107 | -0.397,0.611 | 0.631 |
|  | Female percentage | -3.52 | -16.907,23.947 | 0.696 |
|  | Study duration | -0.025 | -0.099,0.048 | 0.438 |
|  | Baseline CRP | 0.544 | -0.642,1.729 | 0.314 |
|  | CRP change | -0.324 | -3.588,2.939 | 0.773 |
|  | Baseline ESR | 0.057 | -0.189,0.303 | 0.555 |
|  | ACR 20 | 2.163 | -6.597,10.924 | 0.553 |
|  | ACR 50 | 0.911 | -6.843,8.666 | 0.775 |
|  | ACR 70 | -0.139 | -8.664,8.385 | 0.968 |
| Heart failure |  |  |  |  |
|  | Age | 0.003 | -1.115,1.122 | 0.994 |
|  | Female percentage | 3.336 | -45.188,51.861 | 0.867 |
|  | Study duration | 0.002 | -0.1,0.104 | 0.965 |
|  | Baseline CRP | -0.082 | -2.248,2.084 | 0.926 |
|  | ACR 20 | 1.089 | -11.374,13.551 | 0.799 |
|  | ACR 50 | -0.762 | -11.307,9.783 | 0.851 |
|  | ACR 70 | -1.442 | -12.791,9.907 | 0.742 |
|  | DAS28 remission | 0.704 | -13.416,14.824 | 0.903 |
| CV death |  |  |  |  |
|  | Age | 0.158 | -0.146,0.461 | 0.251 |
|  | Female percentage | 6.188 | -18.768,31.144 | 0.552 |
|  | Study duration | -0.032 | -0.093,0.029 | 0.244 |
|  | Baseline CRP | 0.177 | -1.168,1.521 | 0.749 |
|  | Baseline ESR | 0.07 | -0.388,0.528 | 0.66 |
|  | ACR 20 | 2.679 | -26.65,32.009 | 0.79 |
|  | ACR 50 | -0.762 | -15.153,13.63 | 0.89 |
|  | ACR 70 | -0.648 | -15.026,13.73 | 0.907 |
|  | DAS28 remission | 1.632 | -15.357,18.62 | 0.803 |
| All-cause mortality |  |  |  |  |
|  | Age | 0.326 | -0.083,0.734 | 0.106 |
|  | Female percentage | 6.587 | -34.262,47.436 | 0.724 |
|  | Study duration | -0.03 | -0.104,0.044 | 0.387 |
|  | Baseline CRP | 0.272 | -0.957,1.5 | 0.629 |
|  | CRP change | -0.396 | -1.807,1.014 | 0.35 |
|  | Baseline ESR | -0.012 | -0.662,0.639 | 0.958 |
|  | ACR 20 | 9.086 | -12.47,30.643 | 0.328 |
|  | ACR 50 | 1.807 | -11.902,15.517 | 0.758 |
|  | ACR 70 | 0.75 | -13.245,14.746 | 0.9 |
|  | DAS28 remission | -1.474 | -19.11,16.163 | 0.849 |
| 3P-MACE |  |  |  |  |
|  | Age | 0.278 | 0.071,0.486 | **0.011** |
|  | Female percentage | 2.529 | -10.785,15.843 | 0.694 |
|  | Study duration | 0.006 | -0.038,0.049 | 0.794 |
|  | Baseline CRP | 0.53 | -0.137,1.197 | 0.112 |
|  | CRP change | -0.852 | -1.882,0.176 | 0.087 |
|  | Baseline ESR | 0.025 | -0.147,0.196 | 0.757 |
|  | ACR 20 | -0.157 | -5.859,5.545 | 0.953 |
|  | ACR 50 | -1.369 | -6.113,3.375 | 0.546 |
|  | ACR 70 | -1.591 | -6.457,3.276 | 0.495 |
|  | DAS28 remission | -1.317 | -6.967,4.332 | 0.626 |
| 4P-MACE |  |  |  |  |
|  | Age | 0.255 | 0.039,0.471 | **0.023** |
|  | Female percentage | 2.068 | -11.582,15.718 | 0.755 |
|  | study duration | 0.007 | -0.036,0.05 | 0.727 |
|  | Baseline CRP | 0.315 | -0.334,0.964 | 0.321 |
|  | CRP change | -0.674 | -1.63,0.281 | 0.135 |
|  | Baseline ESR | 0.012 | -0.143,0.168 | 0.864 |
|  | ACR 20 | 0.441 | -4.075,4.958 | 0.838 |
|  | ACR 50 | -1.033 | -5.078,3.012 | 0.596 |
|  | ACR 70 | -1.445 | -5.77,2.88 | 0.489 |
|  | DAS28 remission | -0.784 | -5.971,4.402 | 0.753 |
| RA cohort |  |  |  |  |
| MI |  |  |  |  |
|  | Age | -0.093 | -1.469,1.282 | 0.868 |
|  | Female percentage | -0.715 | -11.951,10.521 | 0.876 |
|  | BMI | -0.414 | -6.89,6.063 | 0.566 |
|  | Study duration | 0.036 | -0.208,0.28 | 0.7 |
|  | Diabetes mellitus (%) | -0.357 | -4.181,3.467 | 0.808 |
|  | Hypertension (%) | -0.804 | -6.644,5.036 | 0.691 |
|  | Ever smoking (%) | 1.211 | -5.483,7.905 | 0.518 |
| Stroke |  |  |  |  |
|  | Age | -0.154 | -0.802,0.493 | 0.503 |
|  | Female percentage | 1.682 | -16283,19647 | 0.785 |
|  | Study duration | 0.069 | -0.291,0.429 | 0.495 |
|  | Diabetes mellitus (%) | 0.96 | -11.226,13.147 | 0.767 |
|  | Hypertension (%) | 0.647 | -31.469,32.763 | 0.84 |
| Heart failure |  |  |  |  |
|  | Age | -0.238 | -0.486,0.009 | 0.057 |
|  | Female percentage | -0.687 | -13.881,12.507 | 0.905 |
|  | Study duration | -0.842 | -24.965,23.282 | 0.919 |
|  | Diabetes mellitus (%) | 1.125 | -0.814,3.064 | 0.183 |
|  | Hypertension (%) | 0.505 | -0.805,1.816 | 0.307 |
|  | Dyslipidemia (%) | 1.341 | -24.177,26.859 | 0.625 |
| CV death |  |  |  |  |
|  | Age | 0.005 | -0.577,0.586 | 0.981 |
|  | Female percentage | -3.61 | -11.407,4.188 | 0.237 |
|  | Study duration | 0.44 | -13.798,14.678 | 0.762 |
|  | Diabetes mellitus (%) | 16.036 | -121.468,153.54 | 0.378 |
| All-cause mortality |  |  |  |  |
|  | Age | 0.007 | -0.036,0.05 | 0.701 |
|  | Female percentage | 0.239 | -0.353,0.83 | 0.362 |
|  | Study duration | -0.099 | -0.282,0.085 | 0.186 |
|  | Diabetes mellitus (%) | 0.168 | -1.619,1.954 | 0.819 |
|  | Hypertension (%) | -0.168 | -1.473,1.137 | 0.739 |
|  | Dyslipidemia (%) | 1.298 | -38.413,41.009 | 0.749 |
|  | Ever smoking (%) | 0.593 | -24.797,25.982 | 0.816 |
| Psoriasis |  |  |  |  |
| MI |  |  |  |  |
|  | Age | -0.196 | -0.724,0.331 | 0.44 |
|  | Male percentage | -3.362 | -14.856,8.133 | 0.542 |
|  | BMI | -0.028 | -1.27,1.213 | 0.955 |
|  | Weight | 0.046 | -0.144,0.237 | 0.599 |
|  | Study duration | -0.006 | -0.136,0.124 | 0.92 |
|  | Previous CV event (%) | 7.361 | -57.565,72.286 | 0.386 |
|  | Diabetes mellitus (%) | 39.398 | -227.917,306.714 | 0.591 |
|  | Hypertension (%) | 14.884 | -31.818,61.587 | 0.304 |
|  | Dyslipidemia (%) | 5.557 | -10.384,21.498 | 0.272 |
|  | Baseline CRP | 1.728 | -17.424,20.881 | 0.457 |
|  | PASI50 | 5.24 | -25.365,35.846 | 0.538 |
|  | PASI75 | 2313 | -3.672,8.299 | 0.421 |
|  | PASI90 | 1.378 | -4.101,6.857 | 0.596 |
|  | PASI100 | -0.331 | -9.814,9.153 | 0.94 |
| Stroke |  |  |  |  |
|  | Age | -0.082 | -0.57,0.407 | 0.71 |
|  | Male percentage | -1.97 | -16.649,12.707 | 0.765 |
|  | BMI | 2.038 | -51.634,55.709 | 0.714 |
|  | Weight | 0.012 | -26.593,26.433 | 0.995 |
|  | Study duration | -0.026 | -0.276,0.224 | 0.816 |
| Heart failure |  |  |  |  |
|  | Age | -0.225 | -8.236,7.786 | 0.782 |
|  | Male percentage | 1.472 | -443.471,446.415 | 0.973 |
|  | Study duration | -0.031 | -1.293,1.23 | 0.805 |
| CV death |  |  |  |  |
|  | Age | 0.249 | -1.832,2.331 | 0.756 |
|  | Male percentage | -1.534 | -20.031,16.962 | 0.829 |
|  | Weight | -0.059 | -0.87,0.753 | 0.833 |
|  | Study duration | -0.045 | -0.25,0.161 | 0.579 |
|  | PASI50 | 5.175 | -98.221,108.571 | 0.639 |
|  | PASI75 | 2.748 | -10.739,16.236 | 0.602 |
|  | PASI90 | 2.46 | -10.759,15.678 | 0.633 |
|  | PASI100 | -1.7 | -55.531,52.131 | 0.904 |
| All-cause mortality |  |  |  |  |
|  | Age | -0.093 | -1.15,0.965 | 0.838 |
|  | Male percentage | -1.295 | -15.437,12.847 | 0.83 |
|  | BMI | -1.495 | -8.235,5.245 | 0.441 |
|  | Weight | -0.19 | -0.856,0.475 | 0.471 |
|  | Study duration | -0.045 | -0.22,0.131 | 0.557 |
|  | PASI50 | 5.392 | -97.995,108.779 | 0.627 |
|  | PASI75 | 3.158 | -9.021,15.336 | 0.535 |
|  | PASI90 | 1.207 | -7.849,10.262 | 0.755 |
|  | PASI100 | -2.998 | -18.948,12.952 | 0.629 |
| 3P-MACE |  |  |  |  |
|  | Age | -0.148 | -0.467,0.172 | 0.35 |
|  | Male percentage | -0.062 | -7.383,7.26 | 0.986 |
|  | BMI | -0.075 | -1.127,0.977 | 0.874 |
|  | Weight | 0.077 | -0.078,0.232 | 0.309 |
|  | Study duration | -0.039 | -0.144,0.066 | 0.451 |
|  | Previous CV event (%) | 12.3 | -47.83,72.429 | 0.234 |
|  | Diabetes mellitus (%) | 81.088 | -313.305,475.482 | 0.47 |
|  | Hypertension (%) | 26.621 | -18.474,71.716 | 0.126 |
|  | Dyslipidemia (%) | 9.591 | -5.739,24.92 | 0.115 |
|  | Baseline CRP | 0.064 | -3.529,3.658 | 0.963 |
|  | PASI50 | 5.053 | -10.144,20.249 | 0.447 |
|  | PASI75 | 2.35 | -2.61,7.31 | 0.336 |
|  | PASI90 | 1.491 | -2.929,5.912 | 0.491 |
|  | PASI100 | -1.26 | -8.386,5.866 | 0.712 |
| 4P-MACE |  |  |  |  |
|  | Age | -0.178 | -0.496,0.139 | 0.258 |
|  | Male percentage | 0.034 | -7.129,7.197 | 0.992 |
|  | BMI | -0.098 | -1.078,0.881 | 0.826 |
|  | Weight | 0.067 | -0.084,0.218 | 0.367 |
|  | Study duration | -0.043 | -0.137,0.05 | 0.348 |
|  | Previous CV event | 12.3 | -47.83,72.429 | 0.2334 |
|  | Diabetes mellitus (%) | 81.088 | -313.305,475.482 | 0.47 |
|  | Hypertension (%) | 26.621 | -18.474,71.716 | 0.126 |
|  | Dyslipidemia (%) | 9.591 | -5.739,24.92 | 0.115 |
|  | Baseline CRP | 0.064 | -3529,3.658 | 0.963 |
|  | PASI50 | 4.779 | -8.399,17.957 | 0.409 |
|  | PASI75 | 2.631 | -2.034,7.298 | 0.255 |
|  | PASI90 | 1.437 | -2.559,5.433 | 0.464 |
|  | PASI100 | -1.958 | -8.408,4.493 | 0.529 |
| SLE |  |  |  |  |
| MI |  |  |  |  |
|  | Age | -0.19 | -0.664,0.284 | 0.328 |
|  | Female percentage | -36.86 | -104.74,31.021 | 0.206 |
|  | Study duration | 0.036 | -0.094,0.166 | 0.488 |
|  | SRI response rate | 3.421 | -32.788,39.631 | 0.724 |
| Stroke |  |  |  |  |
|  | Age | 0.094 | -0.439,0.627 | 0.651 |
|  | Study duration | 0.009 | -0.107,0.124 | 0.841 |
|  | Female percentage | 2.225 | -59.454,63.905 | 0.925 |
|  | Baseline BILAG | 0.101 | -4.827,5.03 | 0.837 |
|  | SRI response rate | -2.24 | -117.402,112.922 | 0.846 |
| Heart failure |  |  |  |  |
|  | Age | -0.127 | -0.493,0.24 | 0.416 |
|  | Female percentage | -12.805 | -64.549,38.939 | 0.553 |
|  | Study duration | 0.021 | -0.069,0.111 | 0.578 |
|  | Baseline SLEDAI | -0.842 | -6.709,5.026 | 0.6 |
|  | SRI response rate | -3.005 | -48.166,42.157 | 0.802 |
| All-cause mortality |  |  |  |  |
|  | Age | 0.048 | -0.12,0.215 | 0.538 |
|  | Female percentage | 6.643 | -13.577,36.863 | 0.476 |
|  | Study duration | 0.054 | -0.013,0.121 | 0.099 |
|  | Baseline SLEDAI | -8.4 | -2.381,1.564 | 0.596 |
|  | SRI response rate | -1.611 | -10.53,7.309 | 0.662 |
| 3P-MACE |  |  |  |  |
|  | Age | 0.025 | -0.346,0.396 | 0.875 |
|  | Female percentage | -11.732 | -58.072,34.607 | 0.558 |
|  | Study duration | 0.01 | -0.068,0.087 | 0.773 |
|  | Baseline BILAG | 0.719 | -3.849,5.288 | 0.295 |
|  | Baseline SLEDAI | 2.089 | -10.005,14.183 | 0.272 |
|  | SRI response rate | 8.078 | -18.914,35.07 | 0.411 |
| 4P-MACE |  |  |  |  |
|  | Age | -0.023 | -0.254,0.207 | 0.827 |
|  | Female percentage | -16.975 | -47.285,13.335 | 0.241 |
|  | Study duration | 0.007 | -0.044,0.057 | 0.773 |
|  | Baseline BILAG | 0.543 | -3.751,4.838 | 0.354 |
|  | Baseline SLEDAI | 0.877 | -1.72,3.474 | 0.402 |
|  | SRI response rate | 2.039 | -15.576,19.653 | 0.778 |
|  | Major clinical responses | -1.056 | -156.818,154.706 | 0.945 |
|  | Partial clinical responses | -7.005 | -139.15,125.14 | 0.623 |

*ACR, American College of Rheumatology (ACR) response; Remission is defined as DAS28<2.6. bDMARD, biological disease-modifying anti-rheumatic drug; BILAG, British Isles Lupus Assessment Group; CRP, C-reactive protein; CV, cardiovascular; DAS28, Disease Activity Score in 28 joints; ESR, Erythrocyte Sedimentation Rate; MACE, major cardiovascular event; MI, myocardial infarction; PASI, psoriasis area-and-severity index; PASI 50, achievement of 50% of greater reduction from baseline PASI; SLE, systemic lupus erythematosus; SLEDAI, SLE Disease Activity Index; SRI, SLE Responder Index; RA, rheumatoid arthritis; RCT, randomized controlled trial.*

References in supplement

1. Bejarano V , Quinn M , Conaghan P G , et al. Effect of the early use of the anti–tumor necrosis factor adalimumab on the prevention of job loss in patients with early rheumatoid arthritis[J]. Arthritis Rheum, 2010, 59(10):1467-1474.

2. Burmester GR, Rigby WF, Van Vollenhoven RF, et al. Tocilizumab in early progressive rheumatoid arthritis: FUNCTION, a randomised controlled trial[J]. Annals of the rheumatic diseases, 2015, 75(6).

3. Detert J , Bastian H , Listing J , et al. Induction therapy with adalimumab plus methotrexate for 24 weeks followed by methotrexate monotherapy up to week 48 versus methotrexate therapy alone for DMARD-naive patients with early rheumatoid arthritis: HIT HARD, an investigator-initiated study.[J]. Annals of the Rheumatic Diseases, 2013, 72(6):844-850.

4. Emery P, Keystone E, Tony HP, et al. IL-6 receptor inhibition with tocilizumab improves treatment outcomes in patients with rheumatoid arthritis refractory to anti-tumour necrosis factor biologicals: results from a 24-week multicentre randomised placebo-controlled trial[J]. Ann Rheum Dis, 2008, 67(11): 1516–1523.

5. Emery, Paul, Breedveld, et al. Comparison of methotrexate monotherapy with a combination of methotrexate and etanercept in active, early, moderate to severe rheumatoid arthritis (COMET): a randomised, double-blind, parallel treatment trial.[J]. Lancet, 2008, 372(9636):375-382.

6. Emery P, Bingham CO , Burmester GR , et al. Certolizumab pegol in combination with dose-optimised methotrexate in DMARD-nave patients with early, active rheumatoid arthritis with poor prognostic factors: 1-year results from C-EARLY, a randomised, double-blind, placebo-controlled phase III study[J]. Ann Rheum Dis, 2017, 76(1):96-104.

7. Genovese MC, Becker JC, Schiff M, et al. Abatacept for rheumatoid arthritis refractory to tumor necrosis factor alpha inhibition[J]. N Engl J Med, 2005, 353(11):1114-23.

8. Jones G, Sebba A, Gu J, et al. Extended report:Comparison of tocilizumab monotherapy versus methotrexate monotherapy in patients with moderate to severe rheumatoid arthritis: the AMBITION study[J]. Ann Rheum Dis, 2010, 69(1):88-96.

9. Kavanaugh A, Fleischmann RM, Emery P, et al. Clinical, functional and radiographic consequences of achieving stable low disease activity and remission with adalimumab plus methotrexate or methotrexate alone in early rheumatoid arthritis: 26-week results from the randomised, controlled OPTIMA study[J]. Ann Rheum Dis, 2013, 72(1):64-71.

10. Kay, Jonathan, Matteson, et al. Erratum: Golimumab in patients with active rheumatoid arthritis despite treatment with methotrexate: A randomized, double-blind, placebo-controlled, dose-ranging study[J]. Arthritis Rheum, 2008, 58(4):964-75.

11. Randomized comparison of etanercept with usual therapy in an Asian population with active rheumatoid arthritis: the APPEAL trial[J]. Int J Rheum Dis, 2012, 15(2):188-196.

12. Smolen JS, Beaulieu A, Rubbert-Roth A, et al. Effect of interleukin-6 receptor inhibition with tocilizumab in patients with rheumatoid arthritis (OPTION study): a double-blind, placebo-controlled, randomised trial[J]. Lancet, 2008, 371(9617):987-97.

13. Smolen JS, Kay J, Doyle MK, et al. Golimumab in patients with active rheumatoid arthritis after treatment with tumour necrosis factor alpha inhibitors (GO-AFTER study): a multicentre, randomised, double-blind, placebo-controlled, phase III trial[J]. Lancet, 2009, 374(9685):210-21.

14. Smolen JS , Emery P , Ferraccioli GF , et al. Certolizumab pegol in rheumatoid arthritis patients with low to moderate activity: the CERTAIN double-blind, randomised, placebo-controlled trial[J]. Ann Rheum Dis, 2015, 74(5):843-850.

15. van de Putte LBA, Atkins C, Malaise M, et al. Efficacy and safety of adalimumab as monotherapy in patients with rheumatoid arthritis for whom previous disease modifying antirheumatic drug treatment has failed[J]. Ann Rheum Dis, 2004, 63(5):508-16.

16. Weinblatt ME, Kremer JM, Bankhurst AD, et al. A trial of etanercept, a recombinant tumor necrosis factor receptor:Fc fusion protein, in patients with rheumatoid arthritis receiving methotrexate[J]. N Engl J Med, 1999, 340(4):253-9.

17. Weisman MH, Paulus HE, Burch FX , et al. A placebo-controlled, randomized, double-blinded study evaluating the safety of etanercept in patients with rheumatoid arthritis and concomitant comorbid diseases[J]. Rheumatology, 2007(7):1122-1125.

18. Westhovens R, Yocum D, Han J, et al. The safety of infliximab, combined with background treatments, among patients with rheumatoid arthritis and various comorbidities: a large, randomized, placebo-controlled trial.[J]. Arthritis & Rheumatism, 2014, 54(4):1075-1086.

19. Westhovens R, Robles M, Ximenes AC, et al. Clinical efficacy and safety of abatacept in methotrexate-naive patients with early rheumatoid arthritis and poor prognostic factors[J]. Ann Rheum Dis, 2009, 68(12):1870-7.

20. St Clair EW, van der Heijde DMFM, Smolen JS, et al. Combination of infliximab and methotrexate therapy for early rheumatoid arthritis: a randomized, controlled trial[J]. Arthritis Rheum, 2004, 50(11):3432-43.

21. Yamamoto K, Takeuchi T, Yamanaka H, et al. Efficacy and safety of certolizumab pegol plus methotrexate in Japanese rheumatoid arthritis patients with an inadequate response to methotrexate: the J-RAPID randomized, placebo-controlled trial.[J]. Mod Rheumatol, 2014, 24(5):715-24.

22. Blauvelt A, Prinz JC, Gottlieb AB, et al. Secukinumab administration by pre‐filled syringe: efficacy, safety and usability results from a randomized controlled trial in psoriasis (FEATURE)[J]. Br J Dermatol, 2015, 172(2):484-93.

23. Blauvelt A, Papp KA, Griffiths CEM, et al. Efficacy and safety of guselkumab, an anti-interleukin-23 monoclonal antibody, compared with adalimumab for the continuous treatment of patients with moderate to severe psoriasis: Results from the phase III, double-blinded, placebo- and active comparatore-controlled VOYAGE 1 trial[J]. J Am Acad Dermatol, 2017, 76(3):405-417.

24. Blauvelt A, Leonardi CL, Gooderham M, et al. Efficacy and Safety of Continuous Risankizumab Therapy vs Treatment Withdrawal in Patients With Moderate to Severe Plaque Psoriasis: A Phase 3 Randomized Clinical Trial[J]. JAMA Dermatol, 2020, 156(6): 649-658.

25. Deodhar A, Gottlieb AB, Boehncke WH, et al. Efficacy and safety of guselkumab in patients with active psoriatic arthritis: a randomised, double-blind, placebo-controlled, phase 2 study[J]. Lancet, 2018, 391(10136):2213-2224.

26. Gottlieb A, Menter A, Mendelsohn A, et al. Ustekinumab, a human interleukin 12/23 monoclonal antibody, for psoriatic arthritis: randomised, double-blind, placebo-controlled, crossover trial[J]. Lancet, 2009, 373(9664):633-640.

27. Gordon KB, Langley RG, Gottlieb AB, et al. A Phase III, Randomized, Controlled Trial of the Fully Human IL-12/23 mAb Briakinumab in Moderate-to-Severe Psoriasis[J]. J Invest Dermatol, 2012, 132(2):304-14.

28. Gordon KB, Bruce S, Mark L, et al. Efficacy and safety of risankizumab in moderate-to-severe plaque psoriasis (UltIMMa-1 and UltIMMa-2): results from two double-blind, randomised, placebo-controlled and ustekinumab-controlled phase 3 trials[J]. Lancet, 2018, 392(10148):650-661.

29. Griffiths CEM , Reich K , Lebwohl M, et al. Comparison of ixekizumab with etanercept or placebo in moderate-to-severe psoriasis (UNCOVER-2 and UNCOVER-3): results from two phase 3 randomised trials[J]. Lancet, 2015:541-551.

30. Kavanaugh A, Husni ME, Harrison DD, et al. Safety and Efficacy of Intravenous Golimumab in Patients With Active Psoriatic Arthritis[J]. Arthritis Rheumatol, 2017, 69(11):2151-2161.

31. Krueger GG, Langley RG, Leonardi C, et al. A human interleukin-12/23 monoclonal antibody for the treatment of psoriasis[J]. N Engl J Med, 2007, 356(6):580-92.

32. Lebwohl M, Strober B, Menter A, et al. Phase 3 Studies Comparing Brodalumab with Ustekinumab in Psoriasis[J]. N Engl J Med, 2015, 373(14):1318-28.

33. Leonardi CL , Kimball AB , Papp KA , et al. Efficacy and safety of ustekinumab, a human interleukin-12/23 monoclonal antibody, in patients with psoriasis: 76-week results from a randomised, double-blind, placebo-controlled trial (PHOENIX 1)[J]. Lancet, 2008, 371(9625):1665-74.

34. Mease PJ, Gladman DD, Ritchlin CT, et al. Adalimumab for the treatment of patients with moderately to severely active psoriatic arthritis: results of a double-blind, randomized, placebo-controlled trial[J]. Arthritis Rheum, 2005, 52(10):3279-89.

35. Mease PJ, Fleischmann R, Deodhar AA, et al. Effect of certolizumab pegol on signs and symptoms in patients with psoriatic arthritis: 24-week results of a Phase 3 double-blind randomised placebo-controlled study (RAPID-PsA)[J]. Ann Rheum Dis, 2014, 73(1):48-55.

36. Mease PJ , Mcinnes IB , Kirkham B, et al. Secukinumab Inhibition of Interleukin-17A in Patients with Psoriatic Arthritis.[J]. New England Journal of Medicine, 2015, 373(14):1329-1339.

37. Mease PJ, Rahman P, Gottlieb AB, et al. Guselkumab in biologic-naive patients with active psoriatic arthritis (DISCOVER-2): a double-blind, randomised, placebo-controlled phase 3 trial[J]. Lancet, 2020, 395(10230): 1126-36.

38. Nakagawa H, Niiro H, Ootaki K, et al. Brodalumab, a human anti-interleukin-17-receptor antibody in the treatment of Japanese patients with moderate-to-severe plaque psoriasis: Efficacy and safety results from a phase II randomized controlled study[J]. J Dermatol Sci, 2016, 81(1):44-52.

39. Papp KM, Langley RG, Lebwohl M, et al. Efficacy and safety of ustekinumab, a human interleukin-12/23 monoclonal antibody, in patients with psoriasis: 52-week results from a randomised, double-blind, placebo-controlled trial (PHOENIX 2)[J]. Lancet, 2008, 371(9625):1675-84.

40. Papp KA, Langley RG, Sigurgeirsson B, et al. Efficacy and safety of secukinumab in the treatment of moderate-to-severe plaque psoriasis: a randomized, double-blind, placebo-controlled phase II dose-ranging study.[J]. Br J Dermatol, 2013, 168(2):412-21.

41. Reich K, Armstrong AW, Foley P, et al. Efficacy and safety of guselkumab, an anti-interleukin-23 monoclonal antibody, compared with adalimumab for the treatment of patients with moderate to severe psoriasis with randomized withdrawal and retreatment: Results from the phase III, double-blind, placebo- and active comparator-controlled VOYAGE 2 trial[J]. J Am Acad Dermatol, 2017, 76(3):418-431.

42. Reich K, Papp KA, Blauvelt A, et al. Tildrakizumab versus placebo or etanercept for chronic plaque psoriasis (reSURFACE 1 and reSURFACE 2): results from two randomised controlled, phase 3 trials.[J]. Lancet, 2017, 390(10091):276-288.

43. Furie R, Petri M, Zamani O, et al. A phase III, randomized, placebo-controlled study of belimumab, a monoclonal antibody that inhibits B lymphocyte stimulator, in patients with systemic lupus erythematosus.[J]. Arthritis Rheum, 2011, 63(12):3918–3930.

44. Furie R, Nicholls K, Cheng TT, et al. Efficacy and safety of abatacept in lupus nephritis: a twelve-month, randomized, double-blind study.[J]. Arthritis Rheumatol, 2014, 66(2):379-89.

45. Furie RA, Leon G, Thomas M, et al. A phase 2, randomised, placebo-controlled clinical trial of blisibimod, an inhibitor of B cell activating factor, in patients with moderate-to-severe systemic lupus erythematosus, the PEARL-SC study[J]. Ann Rheum Dis, 2015, 74(9):1667-75.

46. Ginzler EM, Wallace DJ, Merrill JT, et al. Disease control and safety of belimumab plus standard therapy over 7 years in patients with systemic lupus erythematosus[J]. J Rheumatol, 2014, 41(2):300-9.

47. Isenberg D, Gordon C, Licu D, et al. Efficacy and safety of atacicept for prevention of flares in patients with moderate-to-severe systemic lupus erythematosus (SLE): 52-week data (APRIL-SLE randomised trial)[J]. Ann Rheum Dis, 2015, 74(11):2006-15.

48. Isenberg DA, Petri M, Kalunian K, et al. Efficacy and safety of subcutaneous tabalumab in patients with systemic lupus erythematosus: results from ILLUMINATE-1, a 52-week, phase III, multicentre, randomised, double-blind, placebo-controlled study[J]. Ann Rheum Dis, 2016, 75(2):323-31.

49. Khamashta M, Merrill JT, Werth VP, et al. Sifalimumab, an anti-interferon-α monoclonal antibody, in moderate to severe systemic lupus erythematosus: a randomised, double-blind, placebo-controlled study[J]. Ann Rheum Dis, 2016, 75(11):1909-1916.

50. Merrill J, Buyon J, Furie R, et al. Assessment of flares in lupus patients enrolled in a phase II/III study of rituximab (EXPLORER)[J]. Lupus, 2011, 20(7):709-716.

51. Merrill JT, Vollenhoven RFV, Buyon JP, et al. Efficacy and safety of subcutaneous tabalumab, a monoclonal antibody to B-cell activating factor, in patients with systemic lupus erythematosus: results from ILLUMINATE-2, a 52-week, phase III, multicentre, randomised, double-blind, placebo-controlled study[J]. Ann Rheum Dis, 2016, 75(2):332-40.

52. Morand EF, Furie R, Tanaka Y, et al. Trial of Anifrolumab in Active Systemic Lupus Erythematosus [J]. N Engl J Med. 2020 Jan 16;382(3):211-221.

53. Navarra SV, Guzmán RM, Gallacher AE, et al. Efficacy and safety of belimumab in patients with active systemic lupus erythematosus: a randomised, placebo-controlled, phase 3 trial[J]. Lancet, 2011, 377(9767):721-31.

54. Rovin BH, Furie R, Latinis K, et al. Efficacy and safety of rituximab in patients with active proliferative lupus nephritis: The Lupus Nephritis Assessment with Rituximab study[J]. Arthritis Rheum, 2012, 64(4):1215-26.

55. Wallace DJ, Kalunian K, Petri MA, et al. Efficacy and safety of epratuzumab in patients with moderate/severe active systemic lupus erythematosus: results from EMBLEM, a phase IIb, randomised, double-blind, placebo-controlled, multicentre study[J]. Ann Rheum Dis, 2014, 73(1):183-90.

56. Al-Aly Z, Pan H, Zeringue A , et al. Tumor necrosis factor-α blockade, cardiovascular outcomes, and survival in rheumatoid arthritis[J]. Transl Res, 2011, 157(1):10-8.

57. Bili A, Tang X, Pranesh S, et al. Tumor Necrosis Factor α Inhibitor Use and Decreased Risk for Incident Coronary Events in Rheumatoid Arthritis[J]. Arthritis Care Res, 2014, 66(3):355-63.

58. Carmona L , Descalzo M N , Perez-Pampin E , et al. All Cause and Cause-Specific Mortality in Rheumatoid Arthritis are not Greater than Expected when Treated with TNF Antagonists[J]. Ann Rheum Dis, 2007, 66(7):880-5.

59. Curtis JR, Kramer JM, Martin C, et al. Heart failure among younger rheumatoid arthritis and Crohns patients exposed to TNF-alpha antagonists.[J]. Rheumatology, 2007, 46(11):1688-93.

60. Dixon WG , Watson KD , Lunt M, et al. Reduction in the incidence of myocardial infarction in patients with rheumatoid arthritis who respond to anti-tumor necrosis factor alpha therapy: results from the British Society for Rheumatology Biologics Register.[J]. Arthritis Rheum, 2007, 56(9): 2905–2912.

61. Geborek P, Crnkic M, Petersson IF, et al. Etanercept, infliximab, and leflunomide in established rheumatoid arthritis: clinical experience using a structured follow up programme in southern Sweden[J]. Ann Rheum Dis, 2002, 61(9):793-8.

62. Greenberg JD, Kremer JM, Curtis JR, et al. Tumour necrosis factor antagonist use and associated risk reduction of cardiovascular events among patients with rheumatoid arthritis[J]. Ann Rheum Dis, 2011, 70(4):576-82.

63. Jacobsson LTH, Turesson C, Anders Gülfe, et al. Treatment with tumor necrosis factor blockers is associated with a lower incidence of first cardiovascular events in patients with rheumatoid arthritis.[J]. J Rheumatol, 2005, 32(7):1213-8.

64. Cole J , Busti A , Kazi S . The incidence of new onset congestive heart failure and heart failure exacerbation in Veteran's Affairs patients receiving tumor necrosis factor alpha antagonists[J]. Rheumatol Int, 2007, 27(4):369-73.

65. Ljung L, Simard JF, Jacobsson L, et al. Treatment with tumor necrosis factor inhibitors and the risk of acute coronary syndromes in early rheumatoid arthritis[J]. Arthritis Rheum, 2012, 64(1):42-52.

66. Low ASL, Lunt M, Mercer LK, et al. Association Between Ischemic Stroke and Tumor Necrosis Factor Inhibitor Therapy in Patients With Rheumatoid Arthritis[J]. Arthritis Rheumatol, 2016, 68(6):1337-45.

67. Low AS, Symmons DP, Lunt M, et al. Relationship between exposure to tumour necrosis factor inhibitor therapy and incidence and severity of myocardial infarction in patients with rheumatoid arthritis[J]. Ann Rheum Dis, 2017, 76(4):654-660.

68. Morgan CL, Emery P, Porter D, et al. Treatment of rheumatoid arthritis with etanercept with reference to disease-modifying anti-rheumatic drugs: long-term safety and survival using prospective, observational data[J]. Rheumatology, 2014, 53(1):186-94.

69. Setoguchi S, Schneeweiss S, Avorn J, et al. Tumor necrosis factor-α antagonist use and heart failure in elderly patients with rheumatoid arthritis[J]. Am Heart J, 2008, 156(2):336-41.

70. Solomon DH, Rassen JA, Kuriya B, et al. Heart failure risk among patients with rheumatoid arthritis starting a TNF antagonist.[J]. Ann Rheum Dis, 2013, 72(11):1813-8.

71. Solomon DH, Curtis JR, Saag KG, et al. Cardiovascular Risk in Rheumatoid Arthritis: Comparing TNF-α Blockade with Nonbiologic DMARDs.[J]. Am J Med, 2013, 126(8): 730.e9–730.e17.

72. Wolfe F, Michaud K. Heart failure in rheumatoid arthritis: rates, predictors, and the effect of anti-tumor necrosis factor therapy.[J]. Am J Med, 2004, 116(5):305-11.
